# DNGR-1 limits Flt3L-mediated antitumor immunity by restraining tumor-infiltrating type I conventional dendritic cells

**DOI:** 10.1136/jitc-2020-002054

**Published:** 2021-05-12

**Authors:** Francisco J Cueto, Carlos del Fresno, Paola Brandi, Alexis J. Combes, Elena Hernández-García, Alfonso R Sánchez-Paulete, Michel Enamorado, Christian P Bromley, Manuel J Gomez, Ruth Conde-Garrosa, Santos Mañes, Santiago Zelenay, Ignacio Melero, Salvador Iborra, Matthew F. Krummel, David Sancho

**Affiliations:** 1 Centro Nacional de Investigaciones Cardiovasculares Carlos III (CNIC), Madrid, Spain; 2 Hospital la Paz Institute for Health Research (IdiPAZ), Madrid, Spain; 3 Department of Pathology, University of California, San Francisco, California, USA; 4 ImmunoX Initiative, University of California, San Francisco, California, USA; 5 UCSF CoLabs, University of California, San Francisco, California, USA; 6 Department of Immunology, Ophthalmology and ENT, School of Medicine, Universidad Complutense de Madrid, Madrid, Spain; 7 Division of Immunology and Immunotherapy, Center for Applied Medical Research, University of Navarra, Pamplona, Spain; 8 Metaorganism Immunity Section, Laboratory of Host Immunity and Microbiome, National Institute of Allergy and Infectious Diseases, National Institutes of Health, Bethesda, Maryland, USA; 9 Cancer Inflammation and Immunity Group, CRUK Manchester Institute, The University of Manchester, Manchester, UK; 10 Department of Immunology and Oncology, Centro Nacional de Biotecnología/CSIC, Darwin, Madrid, Spain; 11 Instituto de Investigación Sanitaria de Navarra, Pamplona, Spain; 12 University Clinic, University of Navarra, Pamplona, Spain; 13 Centro de Investigación Biomédica en Red Cáncer (CIBERONC), Madrid, Spain

**Keywords:** dendritic cells, immunomodulation, Cancer, immunotherapy, DNGR-1/Clec9a, Flt3L, cDC1, CCL5, maraviroc

## Abstract

**Background:**

Conventional type 1 dendritic cells (cDC1s) are central to antitumor immunity and their presence in the tumor microenvironment associates with improved outcomes in patients with cancer. DNGR-1 (CLEC9A) is a dead cell-sensing receptor highly restricted to cDC1s. DNGR-1 has been involved in both cross-presentation of dead cell-associated antigens and processes of disease tolerance, but its role in antitumor immunity has not been clarified yet.

**Methods:**

B16 and MC38 tumor cell lines were inoculated subcutaneously into wild-type (WT) and DNGR-1-deficient mice. To overexpress Flt3L systemically, we performed gene therapy through the hydrodynamic injection of an Flt3L-encoding plasmid. To characterize the immune response, we performed flow cytometry and RNA-Seq of tumor-infiltrating cDC1s.

**Results:**

Here, we found that cross-presentation of tumor antigens in the steady state was DNGR-1-independent. However, on Flt3L systemic overexpression, tumor growth was delayed in DNGR-1-deficient mice compared with WT mice. Of note, this protection was recapitulated by anti-DNGR-1-blocking antibodies in mice following *Flt3L* gene therapy. This improved antitumor immunity was associated with *Batf3*-dependent enhanced accumulation of CD8^+^ T cells and cDC1s within tumors. Mechanistically, the deficiency in DNGR-1 boosted an Flt3L-induced specific inflammatory gene signature in cDC1s, including *Ccl5* expression. Indeed, the increased infiltration of cDC1s within tumors and their protective effect rely on CCL5/CCR5 chemoattraction. Moreover, *FLT3LG* and *CCL5* or *CCR5* gene expression signatures correlate with an enhanced cDC1 signature and a favorable overall survival in patients with cancer. Notably, cyclophosphamide elevated serum Flt3L levels and, in combination with the absence of DNGR-1, synergized against tumor growth.

**Conclusion:**

DNGR-1 limits the accumulation of tumor-infiltrating cDC1s promoted by Flt3L. Thus, DNGR-1 blockade may improve antitumor immunity in tumor therapy settings associated to high Flt3L expression.

## Background

Cancers are complex systems in which cell types other than malignant cells, such as fibroblasts or immune cells, contribute to the tumor microenvironment (TME) and the outcome of disease.^1–3^ CD8^+^ T cell infiltration constitutes a significant prognostic factor for many cancers,^4^ but CD8^+^ T cell immunity strongly depends on cross-presentation of cell-associated antigens, a role at which *Batf3*-dependent conventional type 1 dendritic cells (cDC1s) excel.^1 5 6^ In fact, the infiltration of tumors by cDC1s is strongly associated with improved overall survival in different types of cancer.^1 2 4^ In addition, we and others described that cDC1s are necessary for immune checkpoint therapy^7 8^ and intratumoral cDC1s correlate with responsiveness to checkpoint inhibitors in patients with cancer.^9^

Besides their cross-presenting ability, cDC1s also outstand at producing IL-12, CXCL9 and CXCL10 and at expressing costimulatory molecules, all of which contribute to effective T cell responses against cancer.^1–3 10^ Indeed, cDC1s mediate T cell priming and the generation of tissue-resident memory CD8^+^ T cells,^11^ a memory T cell subset that contributes to immunity against cancer.^12 13^ cDC1s also contribute to the reactivation of central memory CD8^+^ T cells in the context of tumors.^12^ Thus, it is not surprising that tumors select immune escape mechanisms to avoid cDC1 infiltration. Stabilization of β-catenin in melanoma or liver cancer impedes the production of the cDC1-recruiting CCL4 or CCL5, causing a lack of cDC1 infiltrates within the TME.^3 14^ Also, some tumors express the enzymatic machinery to synthetize PGE_2_, which strongly inhibits NK cell-mediated recruitment of cDC1 into the TME.^4 15^ Efforts to develop efficient strategies to use cDC1s in cancer immunotherapy are being pursued.^16^

DNGR-1 (CLEC9A) is a surface receptor with a highly restricted expression on cDC1s.^17–19^ DNGR-1 recognizes F-actin exposed on necrotic cells and mediates cross-presentation of dead cell-associated antigens,^20–22^ favoring the generation of tissue-resident memory CD8^+^ T cells after viral infections.^11^ Of note, DNGR-1 can also modulate signaling through heterologous receptors, restraining inflammation.^23^ However, whether DNGR-1 plays a role in antitumor immunity remains unexplored.

Flt3L is a growth factor needed for the ontogeny of cDC1s.^24–26^ In mice, exogenous administration of Flt3L expands the dendritic cell (DC) compartment, especially cDC1s.^7 8^ Patients treated with Flt3L displayed a potent expansion of DCs, which can be purified, loaded with tumor antigens and reinfused into patients, resulting in significant responses.^27^ Although final reports for several clinical trials using Flt3L are still expected, the use of Flt3L as a coadjuvant in tumor vaccination showed promising results at improving priming of antitumor cytotoxic T cell responses.^28^ Also, Flt3L can be used in vitro to generate DCs from bone marrow progenitors,^29^ but there is limited knowledge about its immunomodulatory effects.

Here, we have studied the role of DNGR-1 in antitumor immunity. Our data show that, in the context of cancer, DNGR-1 ablation does not impair CD8^+^ T cell cross-priming. Conversely, antitumor immunity is boosted in the presence of Flt3L in DNGR-1-deficient settings, by either genetic ablation or therapeutic blockade. In response to Flt3L therapy, the absence of DNGR-1 favors an inflammatory program of cDC1s that includes an increased expression of *Ccl5*. This drives the recruitment of additional cDC1s, which subsequently leads to enhanced CD8^+^ T cell infiltration within tumors. Consistent with our results, analysis of The Cancer Genome Atlas (TCGA)^30^ shows that coexpression of *FLT3LG* and the *CCL5/CCR5* associates with a more intense cDC1 signature and longer patient survival. The observed DNGR-1-mediated down-modulation of Flt3L-augmented cDC1 infiltration and antitumor immunity thus represents a potential target to improve tumor therapy.

## Methods

### Mouse strains

Mice were bred at the CNIC and UCSF-specific pathogen-free conditions. Mouse strains include C57BL/6 mice, *Clec9a*^gfp/gfp^ mice backcrossed more than 10 times to C57BL/6 (DNGR-1-deficient, B6(Cg)-Clec9atm1.1Crs/J),^20^ *Rag1*^–/–^ mice (B6.129S7-Rag1tm1Mom/J, The Jackson Laboratory), *Rag1*^–/–^*Clec9a*^gfp/gfp^ mice^23^ and *Batf3*^–/–^ mice (B6.129S(C)-Batf3tm1Kmm/J).^6^ To obtain OVA-specific CD8^+^ T cells, we used OTI transgenic mice (C57BL/6-Tg(TcraTcrb)1100Mjb/J) mated with B6-SJL (Ptprca Pepcb/ BoyJ) expressing CD45.1 allele.^11^ We used 7-week-old to 10-week-old mice (males or females) for all experiments in a sex-matched manner. Experiments were repeated two to four times and results were pooled.

### Tumor cell lines

All cell lines (table 1) were cultured in DMEM media supplemented with 10% fetal bovine serum, β-mercaptoethanol, L-glutamine, non-essential amino acids, sodium pyruvate, HEPES and penicillin and streptomycin. Passages were done with trypsin EDTA. One day before injection, cells were passed from confluent plates.

**Table 1:** Tumor cell lines used in this work

Cell line	Description
B16F10	Parental cell line, used between passages 115 and 130
B16Flt3L	Flt3L-expressing B16 cells
B16GFPOVA	Ovalbumin-expressing and GFP-expressing B16 cells
B78mChOVA^1^	Ovalbumin-expressing and mCherry-expressing B78 amelanotic melanoma cells
B16ZsGreen	ZsGreen-expressing B16 cells
MC38	Parental cell line, acquired from Kerafast

All tumors cells were collected with PBS-EDTA from subconfluent plates.

### Inoculation of tumor cells and tumor growth

Cells were washed twice with phosphate-buffered saline (PBS), and 5×10^5^ cells were inoculated subcutaneously in 50 µL apyrogenic PBS (GIBCO) in the flank of isofluorane-anesthetized mice. Tumor measurements were obtained three times a week with an electronic caliper, and tumor size was calculated as the product of the longest dimension and its orthogonal. B16F10, B16ZsGreen, B16GFPOVA and B78mChOVA tumors were allowed to grow for 13 days, when mice were euthanized for analysis. Growth of B16Flt3L and MC38 tumors was allowed until compliance of experimental endpoint conditions, tumor ulceration or tumor size greater than 300 mm^2^.

### In vivo treatments

When indicated, tumor-free mice received a hydrodynamic injection of Flt3L-encoding plasmid (FL) or empty vector. Hydrodynamic injection was performed by intravenous administration into the tail vein of 2 mL room temperature PBS containing 10 µg plasmid in less than 8 s. Where indicated, mice were inoculated with tumors 24 hours after the hydrodynamic injection. Intraperitoneal administration of 100 µg anti-DNGR-1-blocking antibodies (7H11, BioXcell) or isotype control (Sigma) was performed at days 5, 7, 9 and 12 after tumor inoculation. When indicated, 30 mg/kg maraviroc (MVC; Sigma) or vehicle were intraperitoneally administered concurrent with antibody administration. Cyclophosphamide (140 mg/kg; Sigma) was administered following a metronomic regime at days 8 and 14 of tumor development.

### Tissue digestion and flow cytometry

Tumors, lymph nodes (LNs), ears and spleens were collected at indicated time points for flow cytometry analysis. Tumors and ears were finely minced with scissors and digested in 0.5 mg/mL Collagenase IV (Sigma-Aldrich) and 0.2 mg/mL DNase I (Sigma-Aldrich) for 60 min at 37°C with agitation. Spleens were minced and incubated for 15 min with 50 µg/mL Liberase TL (Sigma-Aldrich) and 0.2 mg/mL DNase I (Sigma-Aldrich). All these samples were smashed through a 100 µm mesh filter with a syringe plunger. LNs were disaggregated with forceps and digested in 0.5 mg/mL Collagenase IV (Sigma-Aldrich) and 0.2 mg/mL DNase I (Sigma-Aldrich) for 30 min at 37°C, pipetted at minute 15 of incubation. At the incubation endpoint, one volume of EDTA-containing Fluorescence-activated cell sorting (FACS) buffer was added to all samples. Digested LNs were filtered through a 100 µm mesh.

Single-cell suspensions were stained for 30 min at 4°C with LIVE/DEAD Fixable Aqua Dead Cell Stain Kit (Life Technologies). After washing with PBS, cells were stained in FACS buffer containing anti-CD16/32 (BioXcell), 3% FBS and 0.05% EDTA with the corresponding antibody cocktail for 30 min on ice. Cells were washed again and resuspended in FACS buffer for data acquisition using an LSRFortessa SORP (Becton Dickinson) or a FACSAria Fusion (Becton Dickinson) flow cytometry equipment. The quantification of absolute numbers in immune infiltrates was performed by acquiring a controlled volume of sample corresponding to a fixed weight of digested tissue at a constant speed for the same duration. This method was validated with the use of quantification beads in some of the experiments. Determination of absolute numbers allowed to pool data from the same experimental conditions obtained in independent experiments.

### Purification of antigen-presenting cells

Tumors and tumor-draining lymph nodes (TdLNs) were collected and processed as previously described. Tumor single-cell suspensions were enriched in CD11c^+^ cells with anti-CD11c microbeads (Miltenyi Biotec), for further purification of subpopulations of antigen-presenting cells. Enriched CD11c^+^ cells from tumors were further sorted into MHCII^hi^CD24^+^CD11b^−^CD103^+^ cDC1s, MHCII^hi^CD24^+^CD11b^+^CD103^−^ cDC2s and MHCII^hi^CD24^−^F4/80^+^ TAMs. Single-cell suspensions from TdLNs were enriched in DCs by negative selection using a cocktail of biotinylated antibodies (anti-B220, CD3, NK1.1) and streptavidin microbeads (Miltenyi Biotec). Then, CD11c^med^ MHCII^hi^ CD11b^lo^ CD103^+^ migratory cDC1s, CD11c^med^ MHCII^hi^ CD11b^+^ CD103^−^ migratory cDC2s, CD11c^hi^ MHCII^med^ CD11b^lo^ CD8α^+^ resident cDC1s and CD11c^hi^ MHCII^med^ CD11b^+^ CD8α^lo^ resident cDC2s were sorted. A FACSAria Sorter (Becton Dickinson) and a Synergy 4L Cell Sorter (Sony) were used for purification of antigen-presenting cell subpopulations.

### In vitro coculture of antigen-presenting cells and T cells

Spleens and inguinal LNs from CD45.1^+^ OTI mice were collected for purification of CD8^+^ T cells. After incubation with 50 µg/mL Liberase TL (Sigma-Aldrich) and 0.2 mg/mL DNase I (Sigma-Aldrich), lymphoid organs were smashed through 100 µm mesh filters to obtain single-cell suspensions. CD8^+^ T cells were purified using the EasySep mouse CD8^+^ T cell isolation kit (StemCell). Then, CD8^+^ T cells were stained with CellTrace Violet (Invitrogen, Molecular Probes).

Antigen presenting cells (APCs) were incubated at different cell-cell ratios with 22,500 CellTrace Violet-stained OTI cells for 72 hours. T cell proliferation was quantified by CellTrace Violet dilution by flow cytometry.

### RNA sequencing and qPCR

Tumor-infiltrating cDC1s were collected from pools of two to three mice with bilateral tumors from four experimental conditions (WT mice pretreated with an empty plasmid or FL and *Clec9a*^gfp/gfp^ mice pretreated with an empty plasmid or FL), as previously described using the gating strategy shown in online supplemental figure S1A. cDC1s were directly collected in RLT buffer (Qiagen) containing 10 µmol/mL β-mercaptoethanol and was subsequently purified using the RNeasy Plus Micro Kit (Qiagen). Barcoded RNA-seq libraries were prepared using the Nextera XT DNA Library Preparation Kit (Illumina). RNA sequencing was performed on three pools per condition using the HiSeq 2500 System (Illumina). For quantitative PCR, the High Capacity cDNA Reverse Transcription Kit (Applied Biosystems) was used to generate cDNA. Quantitative PCR was performed in a 7900-FAST-384 instrument (Applied Biosystems) by using the GoTaq qPCR master mix from Promega (Madison, Wisconsin, USA).

10.1136/jitc-2020-002054.supp1 Supplementary data

### RNA-seq data analysis

RNA-seq data analysis was performed by the Bioinformatics Unit of CNIC. Sequencing reads were processed with a pipeline that used FastQC to evaluate their quality, and cutadapt to trim sequencing reads, eliminating Illumina and SMARTer adaptor remains, and to discard reads that were shorter than 30 bp. Resulting reads were mapped against mouse transcriptome GRCm38.91, and gene expression levels were estimated with RNA-Seq by Expectation Maximization (RSEM). Around 88% of the reads from any sample participated in at least one reported alignment. Expression count matrices were then processed with an analysis pipeline that used Bioconductor package limma for normalization (using TMM (trimmed mean of M values) method) and differential expression testing. Changes in gene expression were considered significant if associated to Benjamini and Hochberg adjusted p value <0.1.

The expression profiles of a collection of 2731 genes detected as differentially expressed in any of the six performed contrasts were clustered using k-means with n=10. Several clusters, representing genes with altered expression in control versus mFlex comparisons (independently of genotype), were selected and merged into two metaclusters (MCs).

Comparative pairwise functional analyses of genome-wide transcriptome profiles were performed with gene set enrichment analysis (GSEA) to identify gene sets that had a tendency to be more expressed in either of the conditions being compared. Gene sets, representing functional categories or pathways from the Hallmark were analyzed. Enriched gene sets with family-wise error rate (FWER) <0.1 were considered of interest.

### Analysis of cancer patient data

TCGA gene expression data for breast carcinoma (BRCA), cervical squamous carcinoma (CESC), human skin cutaneous melanoma (SKCM), LUAD, LUSC, HNSC and BLCA datasets was downloaded from the Broad Institute Firehose portal (gdac.broadinstitute.org). Normalized gene expression matrices ending ‘.uncv2.mRNAseq_RSEM_normalized_log2.txt’ were used for downstream analyses. Summarized pan-cancer clinical data was obtained from the SAGE synapse database (syn12026747) and additional information from clinical data files downloaded from cBioPortal. Prior to analysis, all normal tissue samples were removed (those with patient ID ending in 11 or 12). Gene signatures of interest were computed as mean log2-normalized gene expression of signature genes. Patients without overall survival data (OS.time=0 or NA) were removed. Patient stage was annotated as a continuous variable as follows: Stage I=1, Stage II=2, Stage III=3, Stage IV=4. Male patients with BRCA were removed. Patients were stratified according to quantiles by signature scores. Survival analysis was carried out using the survival (3.1–12) and survminer (0.4.6) R packages. We compared the product of the gene expression of FLT3LG and either CCL5 or CCR5 with signature scores for cDC1s *CLNK*, *BATF3*, *XCR1*, *CLEC9A* 4 and *KIT, CCR7, BATF3, FLT3, ZBTB46, IRF8, BTLA* and *MYCL1.*^9^

### Statistical analysis

Prism (GraphPad Software) was used for statistical analysis. Tukey’s range test was used to detect outliers. After validating normal distribution of samples by the Shapiro-Wilk test, two-tailed unpaired Student’s t-test was used to evaluate statistical significance between two conditions or one-way analysis of variance (ANOVA) followed by Fisher’s least significant difference test for three or more conditions. To evaluate the statistical significance of the cross-presentation capacity of antigen-presenting cells and tumor growth curves, two-way ANOVA was performed. Figure legends indicate the number of repetitions for each experiment and the total number of independent mice included (n).

## Results

### DNGR-1 deficiency does not impact cross-presentation of tumor-associated antigens

**Figure 1:**
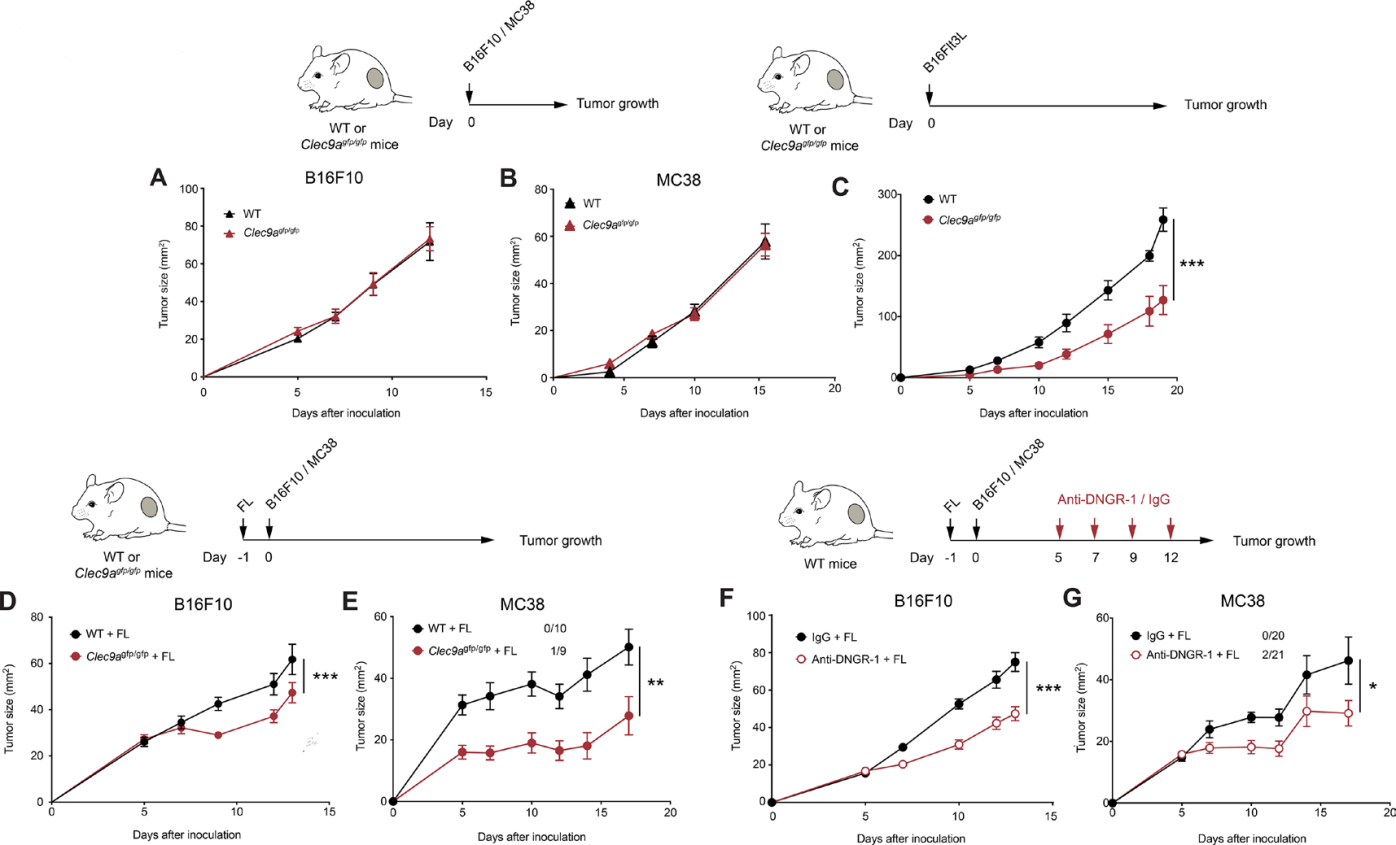
DNGR-1 absence delays tumor growth on Flt3L overexpression. (A, B) B16F10 and MC38 tumors were inoculated subcutaneously in the right flank of WT or *Clec9a*^gfp/gfp^ mice. Growth curves of B16F10 (A) and MC38 (B) tumors. (C) Flt3L-overexpressing B16F10 tumors were inoculated subcutaneously in the right flank of WT (black) or *Clec9a*^gfp/gfp^ (red) mice. The graphs show tumor growth kinetics. (D, E) WT and *Clec9a*^gfp/gfp^ mice were hydrodynamically injected intravenously with a plasmid encoding a secreted form of Flt3L (FL) and 1 day later they were inoculated subcutaneously with B16F10 (D) or MC38 tumors (E). Tumor growth is depicted. (F, G) Tumor growth of B16F10 (F) and MC38 (G) tumors inoculated subcutaneously into WT mice pretreated with FL hydrodynamic injection and receiving intraperitoneal DNGR-1-blocking antibodies (red) or an isotype control (black) at days 5, 7, 9 and 12 of tumor development. (A) Pool of two independent experiments, with n=10 per group. (B) Pool of three independent experiments (n=19 for WT mice and n=20 for *Clec9a*^gfp/gfp^ mice). (C) Pool of two independent experiments, with n=10 per group. (D) Pool of three independent experiments with n=24 for WT mice and n=26 for *Clec9a*^gfp/gfp^ mice. (E) Pool of two experiments with n=10 for WT mice and n=9 for *Clec9a*^gfp/gfp^ mice. (E, G) Numbers following experimental groups indicate number of complete rejections/total number of mice in the experiment. (F) Pool of three experiments with n=22 for mice receiving isotype control and n=24 for mice treated with anti-DNGR-1. (G) Pool of three experiments with n=20 for mice receiving isotype control and n=21 for mice receiving anti-DNGR-1-blocking antibodies. (A–G) Statistical significance was assessed by two-way analysis of variance. All data are shown as mean±SEM. *p<0.05, **p<0.01 and ***p<0.001. WT, wild type.

To assess the role of DNGR-1 in antitumor immunity, we inoculated B16F10 melanoma cells subcutaneously in the flank of WT and DNGR-1-deficient (*Clec9a*^gfp/gfp^) mice. B16F10 melanomas developed similarly in WT and *Clec9a*^gfp/gfp^ mice (figure 1A). To address whether this result was cell line-specific or tumor type-specific, we inoculated WT and *Clec9a*^gfp/gfp^ mice with MC38 colon carcinomas. Again, we found that MC38 tumors grew similarly in WT and *Clec9a*^gfp/gfp^ mice (figure 1B). Following the flow cytometry-gating strategy detailed in online supplemental figure S1A,^1^ we first defined that DNGR-1 expression was restricted to cDC1s among tumor myeloid infiltrates by monitoring the GFP fluorescence derived from the *Clec9a*^gfp/gfp^ knockin cassette (online supplemental figure S1B). The restricted expression of DNGR-1 on cDC1s was further confirmed with anti-DNGR-1 antibodies (online supplemental figure S1C). Then, the analysis of immune cell infiltrates in B16F10 tumors showed that tumors from WT and *Clec9a*^gfp/gfp^ mice contained similar numbers of cDC1s and cDC2s (online supplemental figure S2A). In this line, TdLNs from WT and *Clec9a*^gfp/gfp^ displayed a similar composition in terms of resident and migratory cDC1s and cDC2s (online supplemental figure S2B). These data suggest that DNGR-1 does not affect tumor growth in the steady state.

**Figure 2:**
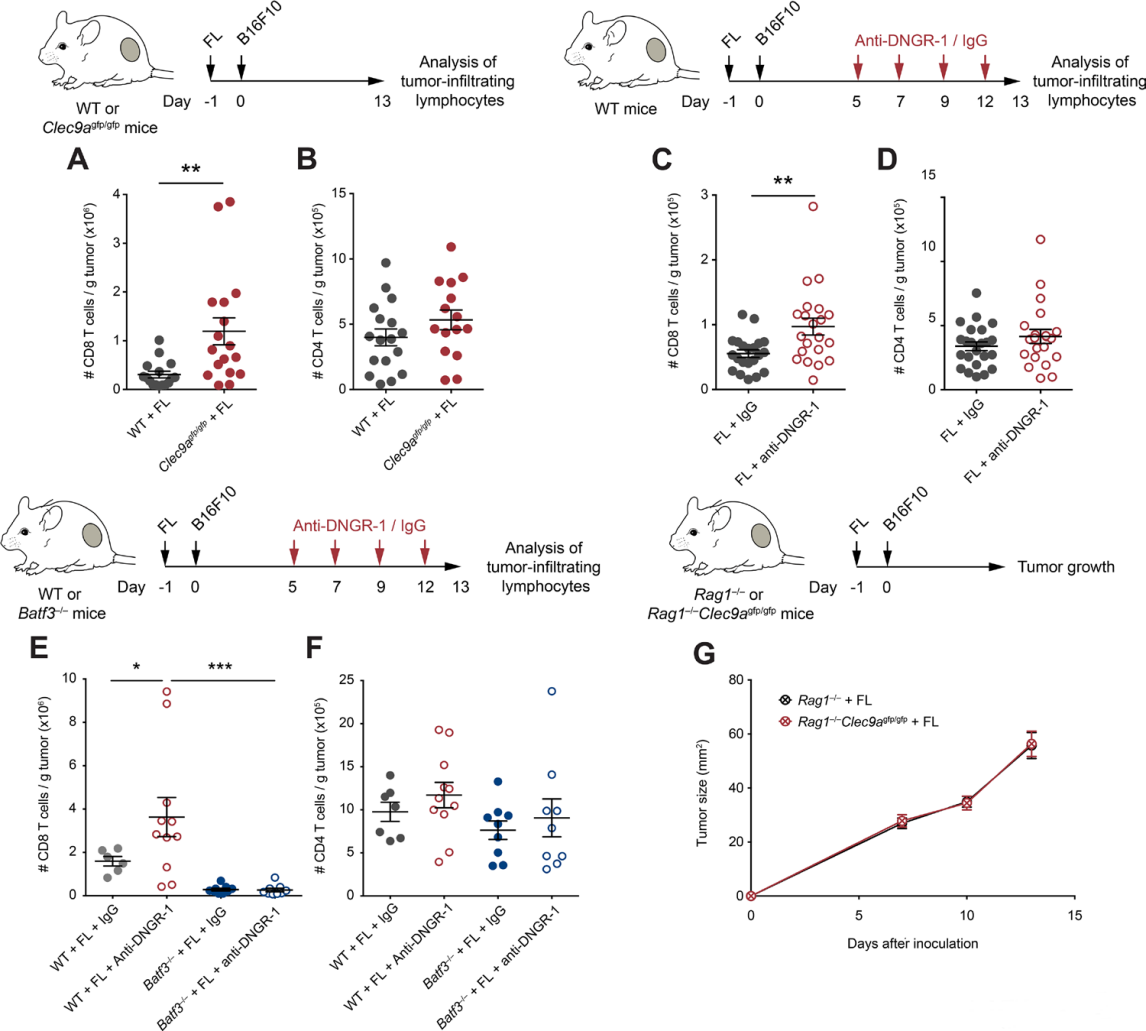
Enhanced CD8^+^ T cell antitumor immunity in Flt3L (FL)-overexpressing DNGR-1-deficient mice. (A, B) WT and *Clec9a*^gfp/gfp^ mice were inoculated with B16F10 tumors subcutaneously in the right flank 1 day after pretreatment with FL. At day 13 after tumor inoculation, tumors were harvested and cell suspensions were analyzed by flow cytometry. Numbers of CD8^+^ (A) and CD4^+^ (B) T cells per gram of B16F10 tumor were quantified. (C–F) WT (C–F) and *Batf3*^–/–^ (E, F) mice were hydrodynamically inoculated intravenously with FL. One day later, B16F10 tumors were injected subcutaneously in the right flank. Mice received intraperitoneal anti-DNGR-1-blocking antibodies or an isotype control at days 5, 7, 9 and 12. At day 13 of tumor development, tumors were collected for flow cytometry analysis of tumor-infiltrating lymphocytes. Numbers of CD8^+^ (A, C, E) or CD4^+^ (B, D, F) T cells per gram of B16F10 tumor are shown. (G) *Rag1*^–/–^ and *Rag1*^–/–^*Clec9a*^gfp/gfp^ mice were pretreated with FL and, 1 day later, B16F10 tumors were inoculated subcutaneously in the right flank. Graph shows tumor growth curves. (A, B) Pool of three experiments with n=17 mice per group. (C, D) Pool of four experiments with n=23 for WT mice treated with isotype control and n=21 for anti-DNGR-1-treated mice. (E, F) Pool of two independent experiment, with n=7 for WT mice receiving isotype control, n=11 for WT mice treated with anti-DNGR-1 antibodies, n=9 for *Batf3*^–/–^ mice receiving isotype control and n=9 for *Batf3*^–/–^ mice receiving anti-DNGR-1 antibodies. (G) Pool of three independent experiments with n=19 for *Rag1*^–/–^ mice and n=14 for *Rag1*^–/–^*Clec9a*^gfp/gfp^ mice. (A–F) Each point represents a single mouse. All data are shown as mean±SEM. Statistical significance was evaluated with Student’s t-test (A–D) and one-way analysis of variance (ANOVA) followed by Fisher’s least significant difference test (E, F). (G) Statistical significance was evaluated with two-way ANOVA. *p<0.05, **p<0.01 and ***p<0.001. WT, wild type.

We next tested whether DNGR-1 contributes to handling of tumor antigens in vivo. With this aim, we injected WT mice with B16ZsGreen tumors. ZsGreen is a green fluorescent protein resistant to lysosomal acid pH, allowing for tracing tumor antigen loading. Next, we treated them with a DNGR-1-blocking antibody intraperitoneally^11 20 23^ or its isotype control, starting at day 5 after tumor inoculation. After 13 days of tumor development, we analyzed the delivery of tumor-derived ZsGreen into DC subsets from TdLNs. We found that DNGR-1 blockade with monoclonal antibodies did not alter the loading of ZsGreen into migratory CD103^+^ or CD103^–^ DCs nor their resident counterparts (online supplemental figure S2C). Also, the frequency of ZsGreen^+^ cells in different DC subsets is consistent with previous studies,^31 32^ with a lower frequency of CD8α^+^ resident DCs bearing tumor-associated antigens (TAAs) (online supplemental figure S2C). We also injected WT and *Clec9a*^gfp/gfp^ mice with B78mCherryOVA tumors to analyze the delivery of mCherry, which is sensitive to lysosomal degradation, in DCs retrieved from TdLNs. In accordance with other studies,^8 32^ we found that migratory cDC1s are the only cells that can retain native tumor-derived mCherry and that the lack of DNGR-1 did not affect it (online supplemental figure S2D).

To directly address a potential role for DNGR-1 in cross-presentation of TAAs, WT and *Clec9a*^gfp/gfp^ mice were injected with B16OVA tumors and, at day 13 after tumor inoculation, we purified antigen-presenting subpopulations from tumors and TdLNs and cocultured them ex vivo with OVA-specific CD8^+^ T (OTI) cells. We observed that tumor-infiltrating or migratory cDC1s were slightly superior than cDC2, tumor-associated macrophages or LN-resident DCs at cross-presenting TAAs, but in a DNGR-1-independent manner (online supplemental figure S2E, F). These data indicate that absence of DNGR-1 does not affect tumor growth, DC infiltrates or the capacity of DCs to deliver TAAs to TdLNs or cross-present them.

### Lack of DNGR-1 delays tumor growth on Flt3L overexpression

Since Flt3L-mediated expansion of Batf3-dependent DCs boosts tumor-specific CD8^+^ T cells,^7^ we tested whether Flt3L effect on cDC1s could be affected by DNGR-1 in the context of tumors. As an initial approach, we tracked the growth of Flt3L-overexpressing B16 tumors (B16Flt3L). Notably, we found that tumor development was delayed in *Clec9a*^gfp/gfp^ compared with WT mice (figure 1C). Because Flt3L expression by cancer cells themselves might differentially regulate immune cell infiltration as soon as tumors become different in size, we performed gene therapy based on the hydrodynamic administration of a secreted Flt3L-encoding plasmid (FL), which results in liver production of the growth factor,^7^ to separately test its effect in parental B16F10 growth. After 14 days, systemic overexpression of FL in tumor-free WT or *Clec9a*^gfp/gfp^ mice did not affect the more primitive monocyte-DC progenitors (online supplemental figure S3A), but expanded common DC progenitors and pre-dendritic cells (pre-DCs) in bone marrow (online supplemental figure S3B, C). Furthermore, FL also drove a prominent expansion of circulating cDC1s and cDC2s (online supplemental figure S3D, E). FL administration promoted the expansion of cDC1s in both spleen (online supplemental figure S3F) and ear skin (online supplemental figure S3G) compared with mice receiving an empty plasmid. Also, cDC2s expanded to a lower extent in both spleen (online supplemental figure S3H) and periphery (online supplemental figure S3I). In both organs, immune populations that do not rely on Flt3L for their expansion, such as neutrophils or macrophages, remained at similar levels in mice treated with FL (online supplemental figure S3J, K). Therefore, FL expanded both cDC1s and cDC2s and their related progenitors. Importantly, FL-mediated DC expansion was equivalent between WT or *Clec9a*^gfp/gfp^ mice (online supplemental figure S3). We then treated WT or *Clec9a*^gfp/gfp^ mice with FL and, 24 hours later, injected them subcutaneously with B16F10 or MC38 tumors. Of note, *Clec9a*^gfp/gfp^ mice displayed a reduced tumor growth (figure 1D, E). These results suggest that DNGR-1 absence limits tumor growth on Flt3L therapy.

As DNGR-1 is a cell surface receptor that possibly senses necrosis in the tumor context, this interaction could be blocked by the use of anti-DNGR-1-blocking antibodies.^11 20 23^ We pretreated WT mice with FL and, 1 day later, inoculated them subcutaneously with B16F10 or MC38 tumors. As tumors grew, we inoculated mice with anti-DNGR-1-blocking antibodies or isotype control. DNGR-1 blockade in the presence of FL delayed tumor growth (figure 1F, G). These results indicate that DNGR-1 blockade combined with Flt3L can be therapeutically targeted as a tumor immunotherapy.

### DNGR-1 dampens antitumor immunity on Flt3L overexpression

To explore the cellular mechanisms underlying protection in DNGR-1-deficient settings following FL treatment, we studied the immune infiltrates of tumors from WT and *Clec9a*^gfp/gfp^ mice pretreated with FL. Consistent with improved antitumor immunity, *Clec9a*^gfp/gfp^ mice contained more CD8^+^ T cells within their tumors (figure 2A), but not more CD4^+^ T cells (figure 2B), when compared with WT controls. Similarly, therapeutic blockade of DNGR-1 resulted in increased CD8^+^ T cell infiltrates in FL-treated mice (figure 2C), but not in CD4^+^ T cells (figure 2D).

Since cDC1s are a key cell type for recruitment of CD8 T cells into tumors,^33^ we evaluated the role of cDC1s in the increased CD8^+^ T cell infiltration of tumors on DNGR-1 blockade, in the presence of FL. For this purpose, we used *Batf3*^–/–^ mice, which have a deficient cDC1 compartment.^6 7^ In contrast with WT mice, the administration of anti-DNGR-1-blocking antibodies did not increase the accumulation of CD8^+^ T cells in *Batf3*^–/–^ mice (figure 2E), suggesting that DNGR-1 blockade mediates its effect through cDC1s. These differences were only observed in CD8^+^ T cells, as CD4^+^ T cell infiltration remained similar in all experimental groups (figure 2F). Furthermore, the adaptive immune compartment was necessary for the protection against tumor growth, as B16F10 tumors grew at similar rates in FL-treated *Rag1^–/–^* and *Rag1^–/–^Clec9a*^gfp/gfp^ mice (figure 2G). Together, these data indicate that, on FL overexpression, DNGR-1 restricts antitumor CD8^+^ T cell responses that are dependent on cDC1s.

### DNGR-1 reduces the accumulation of tumor-infiltrating cDC1s on Flt3L overexpression

**Figure 3:**
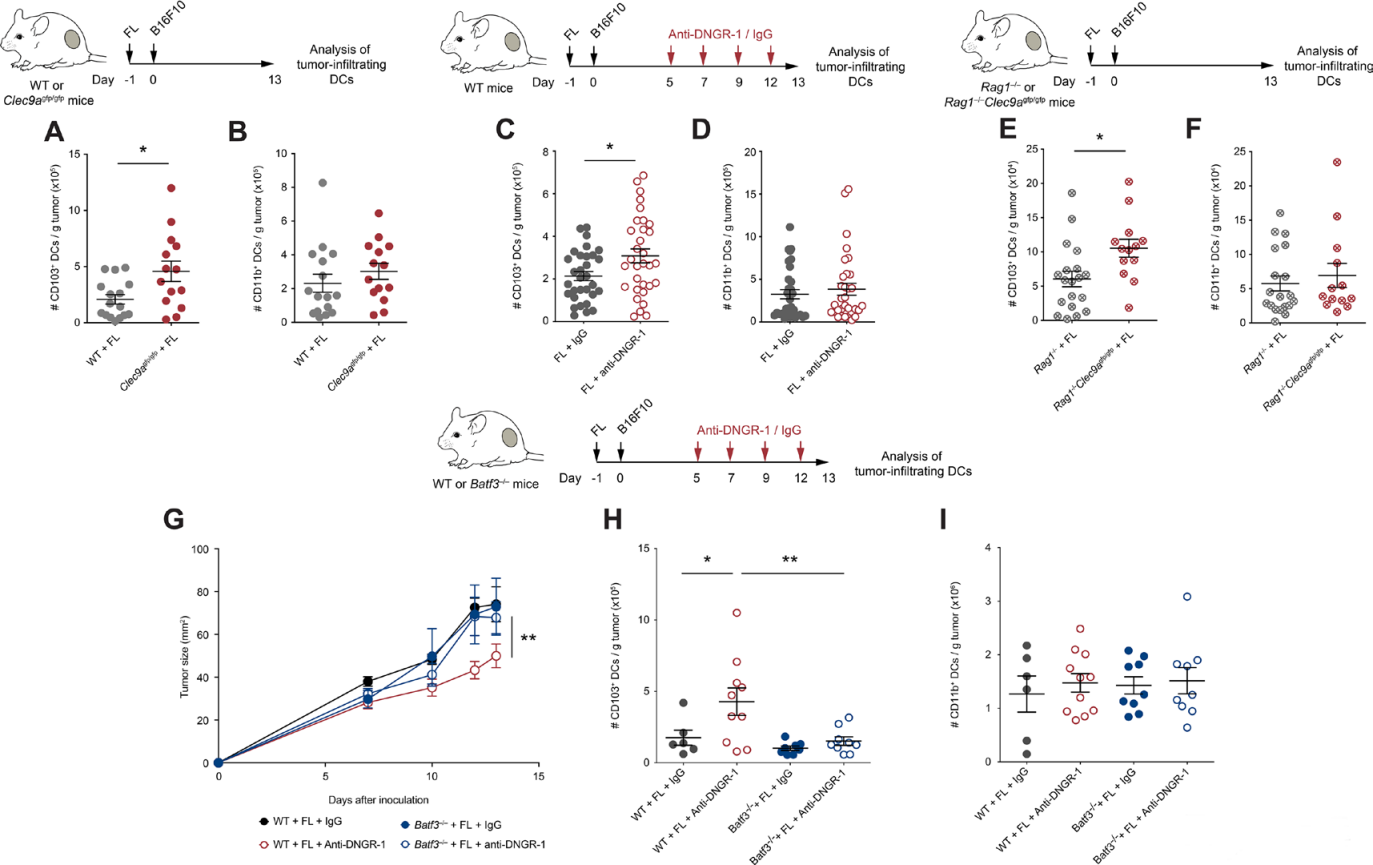
Increased tumor-infiltrating conventional type 1 dendritic cells (cDC1s) control tumor growth in FL-treated DNGR-1-deficient mice. (A, B) WT and *Clec9a*^gfp/gfp^ mice were hydrodynamically injected intravenously with FL. One day later, they were challenged with a subcutaneous inoculation of B16F10 tumors. At day 13 after tumor inoculation, tumors were collected and their immune infiltrates were analyzed by flow cytometry. Numbers of CD103^+^ (A) and CD11b^+^ (B) DCs per gram of tumor. (C–I) WT (C, D, G–I), *Rag1*^–/–^ and *Rag1*^–/–^*Clec9a*^gfp/gfp^ (E, F) and *Batf3*^–/–^ (G–I) mice that had been pretreated with FL 1 day before were inoculated subcutaneously with B16F10 tumors. At days 5, 7, 9 and 12, mice received intraperitoneal 100 µg of anti-DNGR-1-blocking antibodies or an isotype control. At day 13, tumors were collected, and immune infiltrates were analyzed by flow cytometry. Numbers of tumor-infiltrating CD103^+^ (C, E, H) and CD11b^+^ (D, F, I) dendritic cells (DCs) per gram of tumor are shown. (G) Tumor growth curves are depicted. (A, B) Pool of three independent experiments, with n=17 for WT mice and n=14 for *Clec9a*^gfp/gfp^ mice. (C, D) Pool of five independent experiments, with n=32 for each group. (E, F) Pool of three independent experiments, with n=17 for *Rag1*^–/–^ mice and n=13 for *Rag1*^–/–^*Clec9a*^gfp/gfp^ mice. (G–I) Pool of two independent experiments, where n=7 for WT mice injected with isotype control, n=11 for WT mice injected with anti-DNGR-1 antibodies, n=9 for *Batf3*^–/–^ mice receiving isotype control and n=9 for *Batf3*^–/–^ mice injected with anti-DNGR-1 antibodies. (A–F, H, I) Each point represents a single mouse. All data are shown as mean±SEM. (A–F) Student’s t-test was performed to assess statistical significance. (G) Statistical significance was evaluated with two-way analysis of variance (ANOVA). (H, I) Statistical significance was evaluated with one-way ANOVA followed by Fisher’s least significant difference test. *p<0.05 and **p<0.001.

Given the restricted expression of DNGR-1 in cDC1s (online supplemental figure S1B, C), we studied its impact in cDC1 infiltration within tumors. Contrary to the similar DC infiltrates in steady state (online supplemental figure S2A, B), on FL treatment, the accumulation of CD103^+^ cDC1s within tumors in *Clec9a*^gfp/gfp^ mice was boosted compared with WT mice (Figure 3B and online supplemental figure S4A), without affecting tumor infiltration by cDC2s (Figure 3B and online supplemental figure S4B). The increased infiltration of cDC1s, but not cDC2s, within B16F10 tumors was recapitulated in FL-treated WT mice receiving anti-DNGR-1-blocking antibodies (figure 3C, D, online supplemental figure S4C, D). On the contrary, the numbers of the different DC populations at TdLNs remained comparable between WT and *Clec9a*^gfp/gfp^ mice treated with FL (online supplemental figure S4E–H).

To evaluate whether the increased cDC1 infiltration was dependent on the increased CD8^+^ T cell recruitment found on DNGR-1-deficient settings, we analyzed the DC compartment within B16F10 tumors from *Rag1^–/–^* and *Rag1^–/–^Clec9a*^gfp/gfp^ mice treated with FL. Despite having the same size (figure 2G), B16F10 tumors growing in *Rag1^–/–^Clec9a*^gfp/gfp^ mice contained more tumor-infiltrating cDC1s when compared with *Rag1^–/–^* controls (figure 3E), whereas numbers of cDC2s were similar (figure 3F). Thus, the accumulation of tumor-infiltrating cDC1s driven by DNGR-1 deficiency occurs independently of the tumor size and of the adaptive immune compartment. This result suggests that the increased infiltration of cDC1s on FL in DNGR-1-deficient settings precedes and drives adaptive antitumor immunity.

To test whether the effect of DNGR-1 on tumor growth and cDC1 infiltration was directly dependent on cDC1s, we treated WT or *Batf3*^–/–^ mice with FL and inoculated them subcutaneously with B16F10 tumor cells. While WT mice showed delayed tumor growth on DNGR-1 blockade, this protection was lost in *Batf3*^–/–^ mice (figure 3G). Accordingly, *Batf3*^–/–^ mice failed to increase the infiltration of cDC1s within their tumors (figure 3H), while cDC2s remained at similar levels across all experimental conditions (figure 3I). These results indicate that, in the presence of FL, DNGR-1 expression on cDC1s restricts the recruitment of cDC1s and CD8 T cells into the tumor.

### DNGR-1 modulates the gene expression profile induced by Flt3L in tumor-infiltrating cDC1s

**Figure 4:**
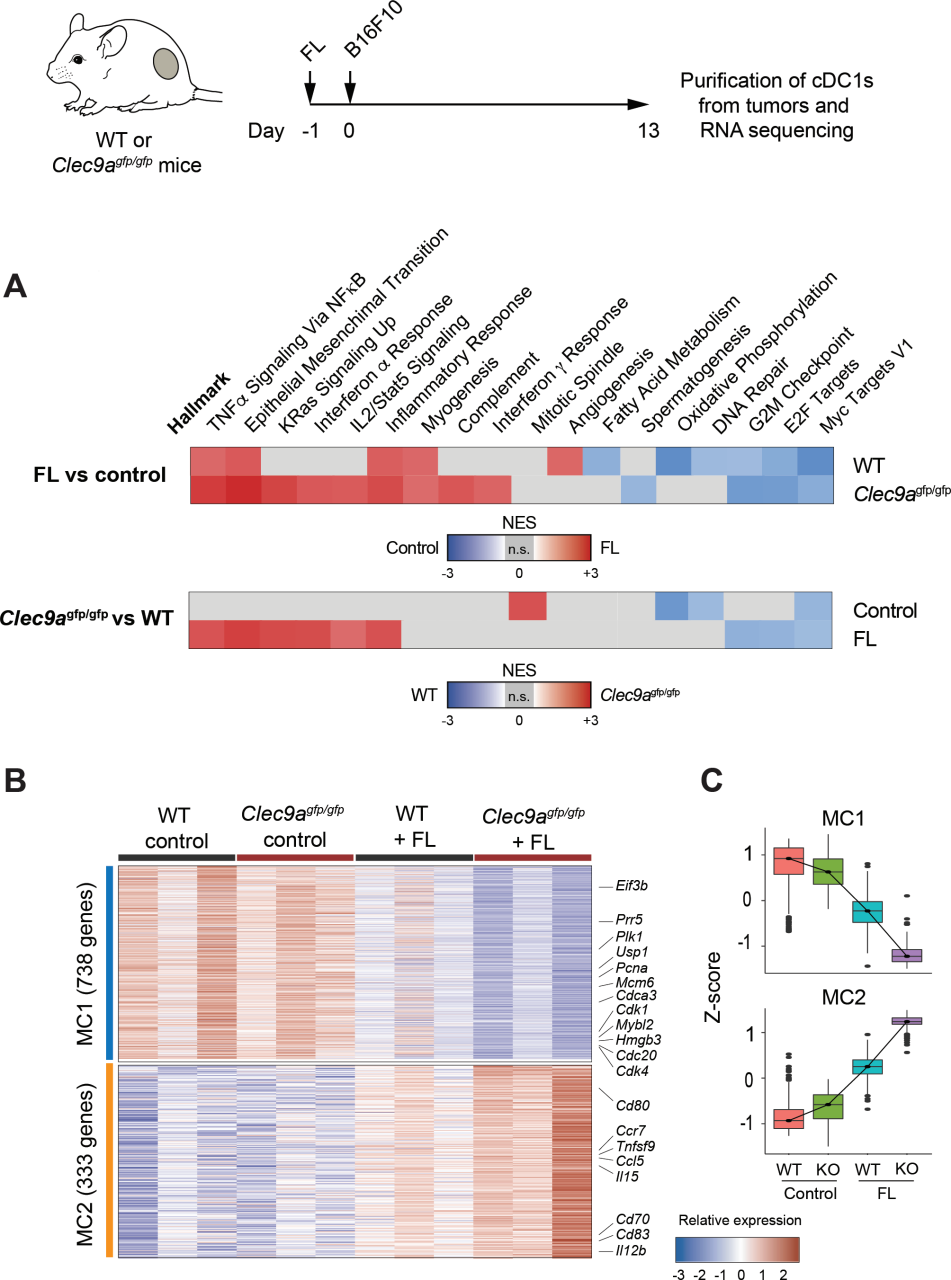
DNGR-1 dampens an activation signature induced by Flt3L (FL) in tumor-infiltrating conventional type 1 dendritic cells (cDC1s). RNA-Seq from CD103^+^ dendritic cells (DCs) purified from day 13 B16F10 tumors grown in WT and *Clec9a*^gfp/gfp^ mice that were hydrodynamically injected intravenously with FL or an empty plasmid. (A) Pairwise gene set enrichment analysis (GSEA). Only significantly enriched gene sets are colored according to their normalized enrichment score (NES). Upper panel: hallmark GSEA of intratumor CD103^+^ DCs from WT and *Clec9a*^gfp/gfp^ mice comparing FL-treated versus empty plasmid (control). Lower panel: hallmark GSEA of intratumor CD103^+^ DCs from FL-treated and empty plasmid (control) comparing WT versus *Clec9a*^gfp/gfp^ mice. Representative enrichment plots are shown in online supplemental figure S5. (B) Gene expression heatmap for metaclusters of interest after k-means clustering of differentially expressed genes in intratumor CD103^+^ DCs across all four conditions. Some genes of interest are highlighted. Three independent experiments were performed with samples from n=2–3 pooled mice per experiment. (C) Z-score for each of the genes belonging to metaclusters 1 and 2 (MC1 and MC2, respectively).

To characterize the molecular mechanisms by which DNGR-1-deficient cDC1s enhance antitumor responses on FL treatment, we performed transcriptomics analysis on cDC1s purified from B16F10 tumors from WT and DNGR-1-deficient mice, either treated with FL or an empty plasmid (control). GSEA revealed seven gene sets modulated by FL in both WT and *Clec9a*^gfp/gfp^ cDC1s, TNFα signaling via NFκB, epithelial mesenchymal transition, inflammatory response, myogenesis, G2M checkpoint, E2F and Myc targets (figure 4A upper panel, online supplemental figure S5A, B). Among these, gene sets related to cDC1 activation, such as the TNFα signaling via NFκB and inflammatory response, were enriched on FL treatment in cDC1s from *Clec9a*^gfp/gfp^ mice compared with those from WT mice (figure 4A lower panel), as well as other immune-related gene sets such as the IFNα response (online supplemental figure S5C). These data suggest that the absence of DNGR-1 enhances some of the activation signatures imprinted by FL.

Out of the total genes differentially expressed in any condition, and according to their behavior across samples, k-means clusters were unified into two MCs of relevance for this study (online supplemental figure S6A). On the one hand, MC1 identified genes whose expression is decreased in WT cDC1s on FL injection, and further downregulated in cDC1s from FL-treated *Clec9a*^gfp/gfp^ mice (figure 4B, C). On the other hand, MC2 comprised clusters 2 and 9 and identified genes that are induced in WT mice on FL injection and are further upregulated in *Clec9a*^gfp/gfp^ cDC1s also on FL (figure 4B, C). Notably, MC2 includes cDC1 costimulatory molecules such as CD80, CD83 or CD70, cytokines such as IL12b or IL15 and chemokine receptors such as CCR7 and chemokines including CCL5. We validated by flow cytometry some relevant features from the RNA-Seq data. Using FL-treated mice that received anti-DNGR-1-blocking antibodies, we confirmed the increase in IL12p40 and CCR7 expression in cDC1s (online supplemental figure S6B, D), but not in cDC2s (online supplemental figure S6C, E). Altogether, these results suggest that DNGR-1 restrains the activation of tumor-infiltrating cDC1s induced by FL.

### Flt3L-induced *Ccl5* expression is enhanced in DNGR-1-deficient cDC1s, favoring their recruitment into the tumor

**Figure 5:**
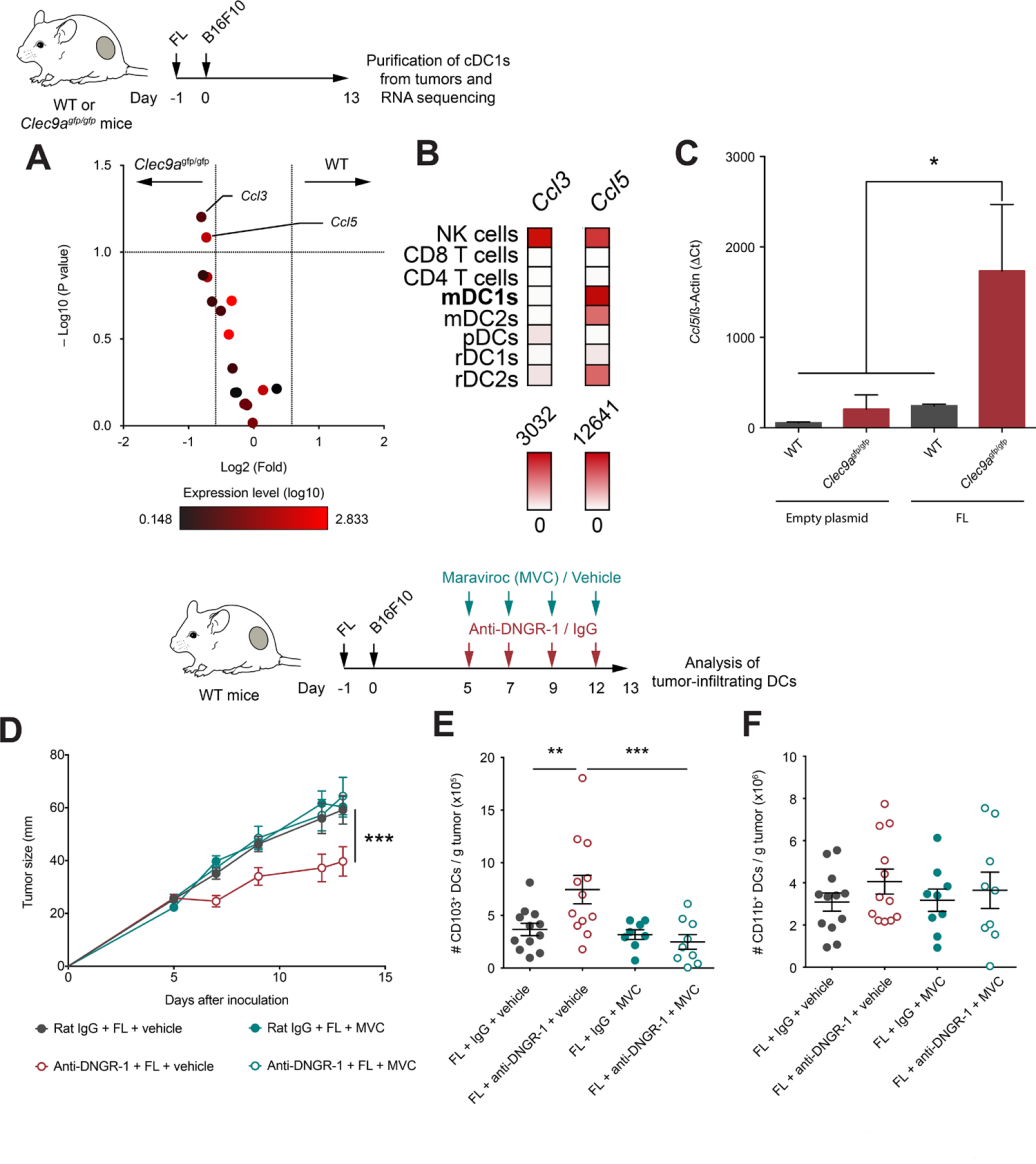
DNGR-1 regulates CCL5/CCR5-mediated infiltration of conventional type 1 dendritic cell (cDC1) in the tumor on Flt3L administration. (A) Volcano plot of chemokines detected in the RNA-seq experiment shown in figure 5. Red color intensity indicates expression level (log10), x-axis indicates fold change of expression in intratumor CD103^+^ dendritic cells (DCs) from WT compared with *Clec9a*^gfp/gfp^ mice and y-axis indicates negative log10 (FDR, p value). (B) Expression profile of *Ccl3* and *Ccl5* across different immune populations from GSE15907. (C) *Ccl5* expression relative to β-actin by qPCR from CD103^+^ DCs purified from 13-day B16F10 tumors grown in WT and *Clec9a*^gfp/gfp^ mice hydrodynamically injected intravenously with FL or an empty plasmid. (D–F) WT mice were pretreated with FL, and after 24 hours, they were inoculated subcutaneously with B16F10 tumors in the right flank. Over the course of tumor growth, mice were treated intraperitoneally at days 5, 7, 9 and 12 with anti-DNGR-1-blocking antibodies or an isotype control. Together with antibody administration, mice received maraviroc (MVC) or vehicle intraperitoneally. Tumor growth kinetics is shown (D). At day 13 after tumor inoculation, tumors were collected for quantification of tumor-infiltrating DCs by flow cytometry. Numbers of CD103^+^ (E) or CD11b^+^ (F) DCs per gram of tumor are shown. (C) Pool of three experiments with n=2–3 mice pooled per experiment. Pool of two independent experiments with n=12 for vehicle groups and n=9 MVC groups. (C–F) All data are shown as mean±SEM. (E, F) Each dot represents a single mouse. (D) Statistical significance was evaluated with two-way analysis of variance (ANOVA). (E, F) Statistical significance was evaluated with one-way ANOVA followed by Fisher’s least significant difference test. *p<0.05, **p<0.01 and ***p<0.001.

Based on our transcriptomic analysis of tumor-infiltrating cDC1s, we focused on chemokine genes as the potential mediators of cDC1s infiltration within the tumor bed. We identified *Ccl5* and *Ccl3* as chemokines induced in DNGR-1-deficient cDC1s versus WT cDC1s on FL administration (figure 5A). However, global *Ccl3* expression was low (figure 5A), suggesting that the highly expressed *Ccl5* could play a more relevant role in cDC1 recruitment. Indeed, analysis of dataset GSE15907^34^ indicates that peripheral migratory mDC1s express *Ccl5* levels comparable to NK cells, while the expression level of *Ccl3* by cDC1s is negligible (figure 5B). Indeed, we confirmed that *Ccl5* was highly expressed in cDC1s from DNGR-1-deficient mice on FL administration compared with cDC1s from WT mice under the same conditions, but also compared with cDC1s from either WT or DNGR-1-deficient mice administered with an empty plasmid (figure 5C).

CCR5 is the chemokine receptor for CCL5, but also the main infection route for the HIV. Several drugs that block the entry of the virus have been developed such as maraviroc (MVC), which also blocks CCR5 signaling and migration in response to CCL5.^35^ To assess whether the CCL5/CCR5 axis is responsible for the enhanced accumulation of cDC1s in tumors from FL-treated mice in DNGR-1-deficient settings, we tested the effect of MVC in tumor growth and DC infiltration. WT mice were treated with FL and injected subcutaneously with B16F10 tumors. During tumor development, mice were treated with either anti-DNGR-1-blocking antibodies or their isotype control and, simultaneously, with MVC or vehicle. Notably, coadministration of MVC reverted the protection provided by anti-DNGR-1 antibodies and FL (figure 5D) and, consistently, impeded the enhanced accumulation of cDC1s on blockade of DNGR-1 (figure 5E). Besides, the infiltration of cDC2s was not affected in any treatment regime (figure 5F). Together, these data indicate that, in the presence of Flt3L, DNGR-1 deficiency in cDC1s boosts CCL5 expression, suggesting that cDC1s may contribute to the recruitment of more cDC1s through the production of CCL5.

### Coexpression of *FLT3LG* and *CCL5*/*CCR5* associates with increased cDC1 score and improved survival in several human cancers

**Figure 6:**
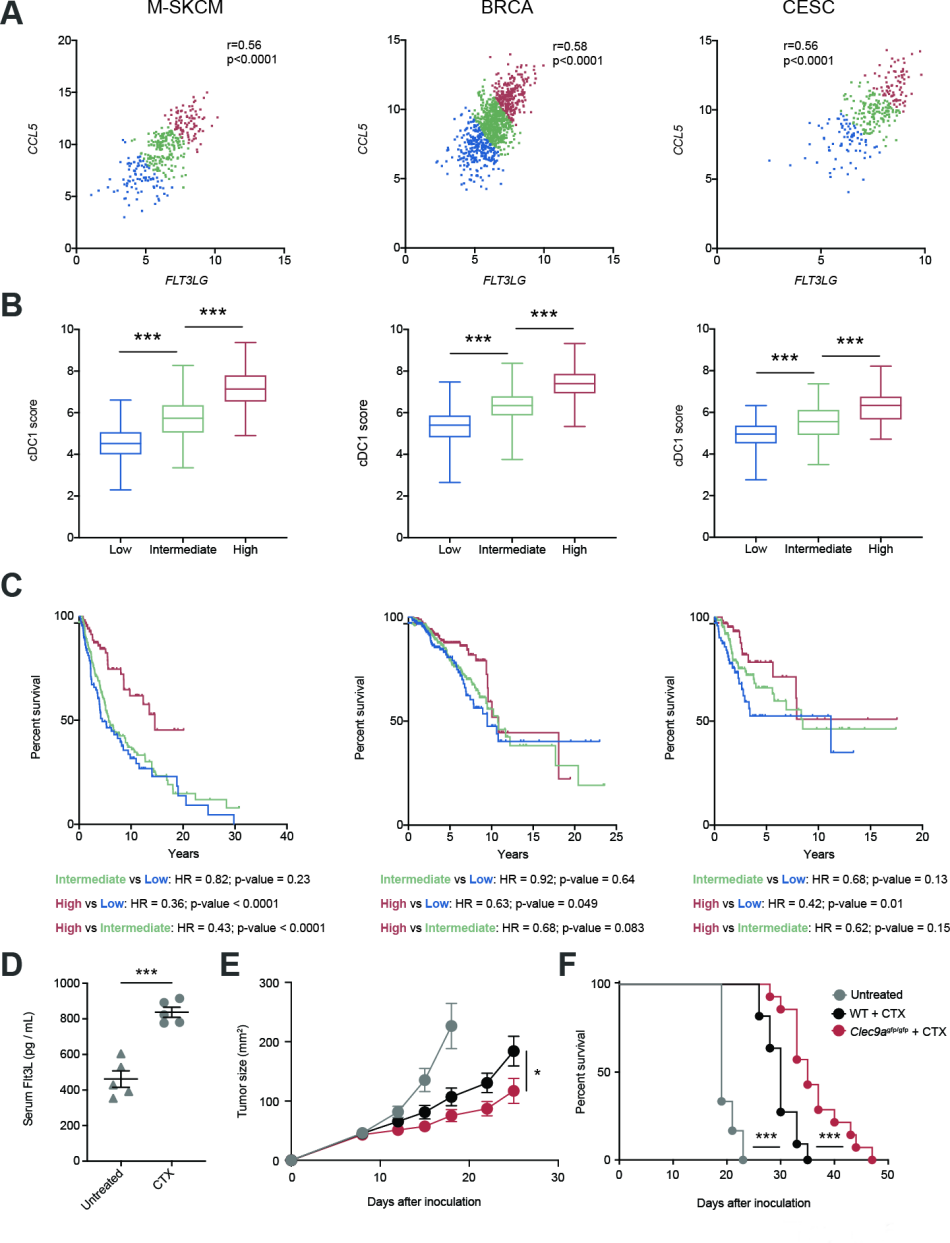
Cooccurrence of *FLT3LG* expression and the *CCL5* associates with increased conventional type 1 dendritic cell (cDC1) score and better survival in several human cancers. (A) Correlation of *FLT3LG* and *CCL5* expression within tumors of metastatic skin cutaneous melanoma (M-SKCM), breast carcinoma (BRCA) and cervical squamous carcinoma (CESC) using data extracted from the The Cancer Genome Atlas. Patients with cancer were assigned an *FLT3LG*/*CCL5* score and sorted into low (first quartile), intermediate (second and third quartiles) and high (fourth quartile). (B) cDC1 score based on expression of *KIT, CCR7, BATF3, FLT3, ZBTB46, IRF8, BTLA* and *MYCL1* as explained in Methods was calculated within tumors from the quartile subcohorts of patients with M-SKCM, BRCA and CESC. (C) Overall survival of the quartile subcohorts of patients indicated in (A). (D–F) B16F10 tumor-bearing WT mice were inoculated with cyclophosphamide (CTX) at days 8 and 14 of tumor development. (D) At day 16, blood was collected and serum levels of FLT3L were quantified. (E, F) B16F10 tumors were inoculated in the flank of WT and *Clec9a*^gfp/gfp^ mice, which were treated with CTX at days 8 and 14. The graphs represent the tumor growth curves (E) and survival curves (F). (A) Each point indicates a patient. r, Pearson correlation coefficient. (B) Whisker plot of cDC1 score for patients with cancer was calculated based on the expression of *KIT, CCR7, BATF3, FLT3, ZBTB46, IRF8, BTLA* and *MYCL1*. Statistical significance was assessed by one-way analysis of variance (ANOVA) followed by Fisher’s least significant difference test. (D) Graph shows the mean of one experiment where each dot represents one mouse. (E, F) These graphs contain a pool of two independent experiments with n=6 for untreated mice, n=11 for WT + CTX and n=14 for *Clec9a*^gfp/gfp^ + CTX. Statistical significance was evaluated by pairwise Mantel-Cox test (C, F), Student’s t-test (D) and two-way ANOVA (E). *p<0.05 and ***p<0.001.

Both *FLT3LG* and *CCL5* have been separately associated with the amount of cDC1s within the TME,^4 9^ but based on our recent findings we hypothesized that the interplay of both factors might impact the infiltration of cDC1s into human cancers and, thus, affect their prognosis. For this reason, we generated an *FLT3LG*/*CCL5* expression signature for patients from TCGA and stratified them into low (first quartile), intermediate (second and third quartiles) and high (fourth quartile). Notably, we found a strong correlation of *FLT3LG* and *CCL5* expression in the case of metastatic human skin cutaneous melanomas (M-SKCM), BRCAs and CESCs (figure 6A). In addition, we calculated a cDC1 score based on the gene expression signature based on expression of *KIT, CCR7, BATF3, FLT3, ZBTB46, IRF8, BTLA* and *MYCL1* as explained in Methods and described by Barry *et al*.^9^ The cDC1 signature score progressively increased with the *FLT3LG*/*CCL5* expression signature (figure 6B). Importantly, patients that coexpressed high transcript levels of *FLT3LG* and *CCL5* experienced longer overall survival compared with patients bearing an intermediate and low expression of the *FLT3LG*/*CCL5* signature (figure 6C). These data support the involvement of CCL5 in cooperation with FLT3LG in the cDC1 infiltration and antitumor response.

Of note, when we replaced *CCL5* by *CCR5* in the analysis, the behavior was similar. *FLT3LG* expression also correlated with *CCR5* expression within tumors from patients with M-SKCM, BRCA or CESC (online supplemental figure S7A). Segregating patients into low, intermediate and high *FLT3LG/CCR5* expressors based on quartiles as indicated above also identified a correlation between the *FLT3LG/CCR5* signature and the cDC1 signature score (online supplemental figure S7B) and an association of high expression of the *FLT3LG*/*CCR5* signature with a better overall survival (online supplemental figure S7C). To compare the predictive power of our *FLT3LG/CCL5* or *FLT3LG/CCR5* signatures with previously described cDC1 gene signatures,^4 9^ we selected the highest and lowest quartiles of patients with TCGA with M-SKCM, BRCA and CESC according to Böttcher *et al*^4^ (online supplemental figure S7D) or Barry *et al*^9^ (online supplemental figure S7E) and analyzed their prognostic power. We observed that the HR and p value of *FLT3LG*/*CCL5* or *FLT3LG/CCR5* signatures were similar to those of cDC1 scores and even better in the case of M-SKCM (online supplemental figure S7F). These data indicate that the coexpression of *FLT3LG* and both *CCL5* and *CCR5* is a simplified biomarker for cDC1 tumor score and overall survival in several types of human cancers with similar or even improved predictive power than whole cDC1 signatures.

Given that some chemotherapies, such as cyclophosphamide, are linked to increased expression of Flt3L,^36^ we hypothesized that DNGR-1 absence could enhance tumor therapy in these settings. First, we confirmed that the administration of the chemotherapeutic agent cyclophosphamide increased serum levels of Flt3L (figure 6D). Of note, under these pharmacological settings of enhanced Flt3L concentration, the absence of DNGR-1 synergized with the intrinsic effect of cyclophosphamide, delaying B16 tumor growth (figure 6E) and extending survival (figure 6F). These data represent a proof-of-concept for a potential chemo-immunotherapy based on the administration of an Flt3L-inducing drug such as cyclophosphamide in combination with DNGR-1 deficiency, as a boost for the intrinsic antitumor effect of the chemotherapy.

## Discussion

Radiation therapy and chemotherapeutic agents can induce different forms of cell death that are immunogenic and boost antitumor immune responses.^37^ Moreover, necrosis occurs spontaneously in untreated cancer due to nutrient deprivation, associating with aggressiveness and worse prognosis in many cancer types.^38^ This suggests that detection of dead cancer cells by the immune system may lead to immunogenic or immunosuppressive outcomes depending on the scenario. DNGR-1 is a C-type lectin receptor highly restricted to cDC1s that senses necrosis.^20–22^ The infiltration of cDC1s within the TME has been revealed as a biomarker for improved overall survival in different types of cancer.^1 2 4^ This may be linked to their ability to cross-prime antitumor CD8^+^ T cells.^7 8^

Since DNGR-1 mediates cross-presentation of dead cell-associated antigens,^20–22^ which are abundant in the TME, we hypothesized that DNGR-1 functions could contribute to antitumor immunity. On the contrary and to our surprise, we find here that DNGR-1 is redundant for cross-presenting tumor antigens in the steady state, contrary to other molecules such as Sec22b or Wdfy4.^39 40^ Sec22b and Wdfy4 broadly regulate cross-presentation of bead-associated or soluble antigens,^39 40^ while DNGR-1 is only required for the cross-presentation of necrotic cargo.^20 41^ One of the mechanisms involved in cross-presentation relies on the capacity of antigen-presenting cells to prevent degradation of native proteins within their phagosomes, maintaining a high phagosomal pH through the GTPase Rac2.^5^ This was also demonstrated in the tumor context using tumors labeled with fluorescent proteins.^8 32^ In agreement with this, our results show that only migratory cDC1s maintain detectable levels of mCherry, but its stability does not depend on DNGR-1, as shown for necrosis-associated antigens.^41^ In addition, apart from cDC1s, we find that cDC2s, which do not express DNGR-1, can also cross-present TAAs to CD8^+^ T cells, in line with previous reports.^1 7^ This process could be mediated by cross-presentation per se involving the intracellular pathway, or by cross-dressing of cancer cell membrane fragments.^42^

While antitumor immunity in the steady state is not affected by the absence of DNGR-1, we find that genetic ablation or therapeutic blockade of DNGR-1 in the presence of Flt3L results in delayed tumor growth. This correlates with an increased infiltration of tumors by CD8^+^ T lymphocytes, which constitutes a good prognosis marker for patients with cancer.^1–3^ Of note, CD8^+^ T cell infiltration does not rely on the abundance of immunogenic peptides in the tumor, but rather on the amount of tumor-infiltrating cDC1s.^43^ Indeed, cDC1s express high levels of CXCL9 and CXCL10, the main factors governing the recruitment of effector CD8^+^ T cells into the TME.^33^ We find that the accumulation of CD8^+^ T cells indeed relies on Batf3-dependent cDC1s. In addition, on overexpression of Flt3L, the absence or blockade of DNGR-1 results in increased numbers of infiltrating cDC1s, but not cDC2s within tumors. It is well established that cDC1s are necessary not only for attracting CD8^+^ T cells^33^ but also for efficient antitumor immunity^6^ and the in situ stimulation of T cell responses.^3 33 43^

The paucity of cDC1s makes the in vivo administration of Flt3L, a growth factor that promotes proliferation, differentiation and survival of DCs, a suitable strategy to increase the availability of this cell type.^29^ But there is limited knowledge about its immunomodulatory effects on cDC1s. Patients treated with Flt3L displayed a potent expansion of DCs, which could be purified, loaded with tumor antigens and reinfused.^27^ Although final reports for several clinical trials using Flt3L are still expected, the use of Flt3L as a coadjuvant in tumor vaccination reported promising results at improving priming of antitumor cytotoxic T cell responses.^28^ Our results suggest that DNGR-1 blockade holds therapeutic potential in this context. Furthermore, some of the current cancer treatments can boost the overproduction of endogenous Flt3L, such as radiation therapy and some chemotherapeutic agents.^36^ Indeed, our preclinical data establish the proof-of-principle for a combined chemo-immunotherapy based on cyclophosphamide in a DNGR-1-deficient setting.

Mechanistically, we show that Flt3L increased activation hallmarks on tumor-infiltrating cDC1s. These gene sets are further upregulated in cDC1s from Flt3L-treated *Clec9a*^gfp/gfp^ mice, suggesting that DNGR-1 signaling dampens heterologous signaling via Flt3 (CD135). DNGR-1 contains a hemITAM intracellular motif that signals via Syk but does not drive DC activation.^20 41^ ITAMs can acquire an inhibitory conformation (ITAMi) that also signals through phosphatases and limit other signaling pathways, as in the case of FcγRIIA and Mincle.^44 45^ We found that DNGR-1 signaling can also trigger the activation of SHP1, a phosphatase that inhibits signaling through heterologous receptors.^23^ In some contexts of sterile or infectious tissue damage, DNGR-1 mediates tolerance to disease by inhibiting heterologous pathways triggered by activating receptors, dampening the infiltration of innate immune cells.^23^ Here, DNGR-1 may act as an immune checkpoint by dampening the response of differentiated tumor-infiltrating cDC1s to Flt3L, thus restraining antitumor immunity. Since DNGR-1 is expressed in DC progenitors,^46^ we cannot rule out that some effects could be imprinted during cDC1 differentiation, although we observed that the numbers of DC-committed progenitors were comparable between WT and DNGR-1-deficient mice, even in the presence of FL overexpression that induced their specific expansion.

In the context of Flt3L stimulation, DNGR-1 deficiency boosts the activation of cDC1s. We observed increased expression of genes encoding for costimulatory molecules such as *Il12b*, *Il15* or *Cd70*.^16^ Besides, we found increased expression levels of *Ccr7*, a LN-homing receptor,^32^ but this did not impact the accumulation of migratory cDC1s in the draining LN. While it is likely that all of these activation markers contribute to antitumor immunity, we focused our attention on *Ccl5.* CCL5 is a chemokine whose production in the TME has been previously associated to NK cells or tumor cells themselves. Here, we identify cDC1s as an important source of CCL5, which is further upregulated in *Clec9a*^gfp/gfp^ mice on Flt3L administration. We found that MVC, a CCR5 (chemokine receptor for CCL5) antagonist, prevents the increased recruitment of cDC1s to the tumor on DNGR-1 blockade in the presence of Flt3L. These results concur with previous data showing that the CCL5/CCR5 axis is essential for the recruitment of cDC1s to the TME.^4 14^ In accordance with this, in human melanomas with stabilized β-catenin, tumor-infiltrating cDC1s are strongly reduced, as well as the numbers of CD8^+^ T cells which is due to the lack of tumor-derived CCL4, another ligand for CCR5.^3^ Similarly, β-catenin stabilization in hepatocellular carcinoma results in exclusion of cDC1s from TME, which can be recuperated by forcing *Ccl5* expression on β-catenin-stabilized tumor cells.^14^ Our work highlights that activated tumor-infiltrating cDC1s can also be key producers of CCL5 in DNGR-1-deficient settings in the presence of Flt3L, able to enhance the infiltration of more cDC1s.^31 34 47^

However, the role of CCL5 in antitumor immunity remains controversial. Blockade of CCR5 signaling prevents tumorigenic activity by decreasing inflammation.^48^ This phenomenon can also be observed in patients with colorectal cancer liver metastases, where CCR5 inhibition diminishes cancer progression.^49^ In contrast, the overexpression of CCL5 in different mouse models delays tumor growth.^4^ To explore the potential clinical relevance of our results, we analyzed the expression of *CCL5* and *CCR5* in combination with *FLT3LG* in the TCGA database in relation to a cDC1 score defined by Barry *et al*^9^ and overall survival in patients with metastatic melanoma, breast cancer and cervical cancer. This analysis identifies *CCL5*/*FLT3LG* or *CCR5*/*FLT3LG* coexpressors as the subset with the highest cDC1 score and with a more extended overall survival. This suggests that the concomitant activation of the CCL5/CCR5 axis in the context of Flt3L-based immunotherapy may enhance antitumor immunity in patients with cancer, and this could be potentiated by DNGR-1 blockade. Our data suggest that CCL5 has a tumor-restricting effect by promoting infiltration of antitumor immune cells when coexpressed with high Flt3L levels. Of note, our analysis of the TCGA indicates that the prognostic power of *FLT3LG*/*CCL5* or *FLT3LG*/*CCR5* signatures compares with cDC1 scores and even outperform them in the case of patients with metastatic cutaneous melanoma. Taken together, these data suggest that efficient antitumor immunity requires the co-occurrence of molecular cues that define recruitment of cDC1s as well as conditioning of the TME.

Since DNGR-1 is a surface receptor, it is an easily accessible target for cancer immunotherapy. Indeed, the selective high expression of DNGR-1 in cDC1s has been previously used as a target for delivering cancer antigens to cDC1.^17^ Moreover, although we have found no role for DNGR-1 in tumor antigen cross-presentation in the steady state, there may be potential cancer immunotherapy contexts where the role of DNGR-1 in tumor dead cell antigen cross-presentation may prevail, favoring antitumor immunity. However, our results show that caution is needed as DNGR-1 restricts cDC1 activation in the TME in certain contexts, which could pose a limitation on therapeutic strategies based on the potentiation of cDC1 activity. DNGR-1 blockade or genetic ablation in FL-treated mice delays tumor growth, increasing tumor infiltration by cDC1s and CD8^+^ T cells. TCGA visibly indicates that cDC1 signatures within the TME strongly favor prognosis in patients with cancer, so that cDC1 infiltration should be pursued. Inducing expression of specific chemokines that attract cDC1s by tumor-infiltrating immune cells may be one strategy. We show here that cDC1 infiltration and immune-promoting capacity can be co-attained by combined Flt3L administration and neutralizing DNGR-1 antibodies to elicit production of CCL5, thereby leading to more effective antitumor immune responses.

## Data Availability

Data are available in a public, open access repository. The gene expression data generated in this study is available at GEO: GSE145534.
