# Supplementary material for: DNGR-1 limits Flt3L-mediated antitumor immunity by restraining tumor-infiltrating type I conventional dendritic cells
Source: J Immunother Cancer. 2021 May 12;9(5):e002054. doi: 10.1136/jitc-2020-002054 (PMC8118081; doi:10.1136/jitc-2020-002054)
Supplement: Supplementary data [file jitc-2020-002054supp001.pdf]

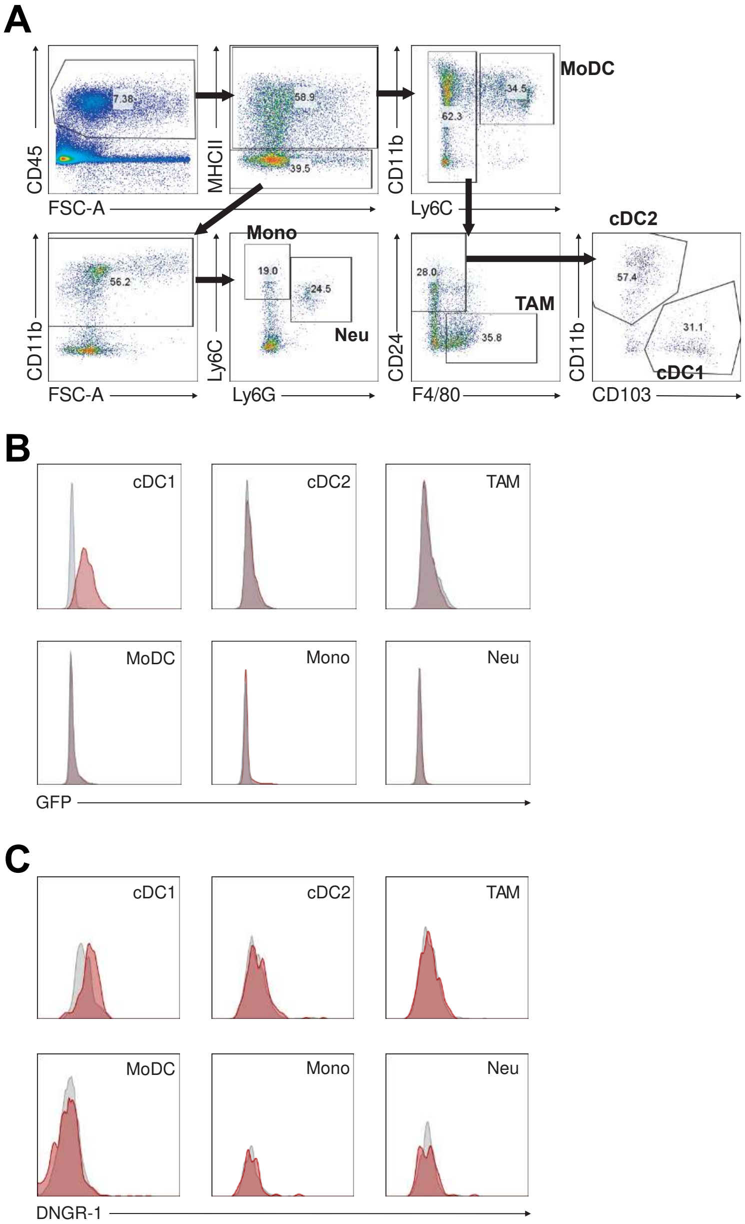


**Figure S1. Flow cytometry gating strategy for myeloid infiltrates of B16F10 tumors.**
(**A**) B16F10 tumors were inoculated s.c. in the right flank of WT mice and single cell suspensions of the tumor were analyzed by flow cytometry 13 days later for the indicated markers. A representative experiment is shown.
(**B**) B16F10 tumors were inoculated s.c. in the right flank of WT (grey histograms) and *Clec9a*$^{gfp/gfp}$ (red) mice and single cell suspensions of the tumor were analyzed by flow cytometry 13 days later as indicated in Figure S1. GFP fluorescence indicative of DNGR-1 expression for the indicated immune cell subset is depicted. A representative experiment is shown.
(**C**) B16F10 tumors were inoculated s.c. in the flank of WT (red histograms) and *Clec9a*$^{gfp/gfp}$ (grey) mice. 13 days later, tumor immune infiltrates were analyzed by flow cytometry for DNGR-1 expression using anti-DNGR-1 antibodies. A representative experiment is shown.

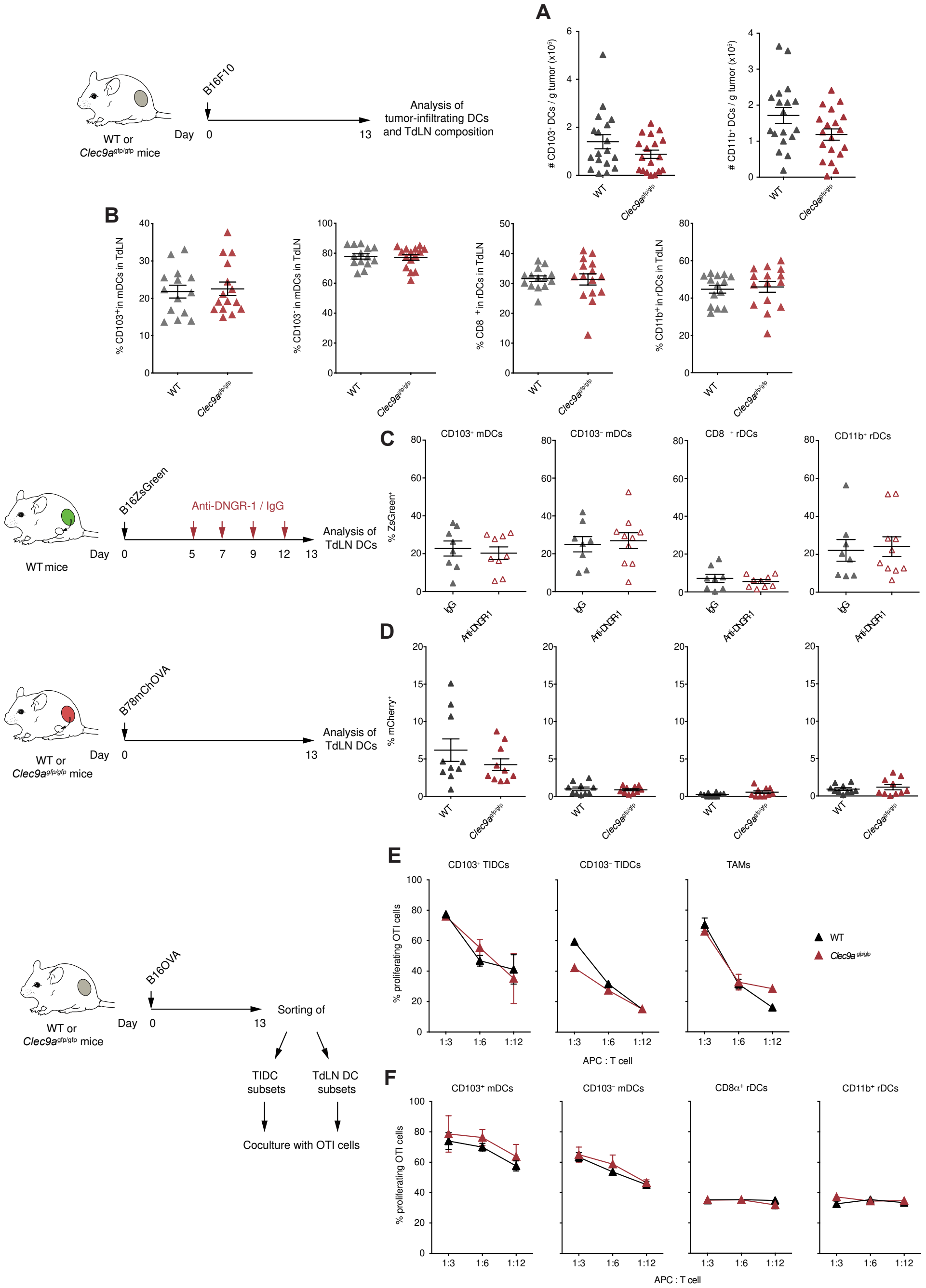


**Figure S2. DNGR-1 is not essential for tumor antigen cross-presentation.**

(**A,B**) WT and *Clec9a*$^{gfp/gfp}$ mice were inoculated s.c. in the right flank with B16F10 tumors. At day 13, tumors and TdLNs were harvested and cell suspensions were analyzed. Quantification by FACS of CD103$^{+}$ and CD11b$^{+}$ DCs per gram of tumor (A), and percentage of TdLN cDC1s and cDC2s among migratory and resident DCs (B). (**C**) B16ZsGreen tumors were inoculated s.c. in the right flank of WT mice. Blocking anti-DNGR-1 antibody was administered i.p. at day 5, 7, 9 and 12 of tumor development. Percentage of

ZsGreen$^+$ cells within different subsets of DCs (mDCs: migratory DCs; rDCs: resident DCs) was analyzed by flow cytometry at the tumor draining lymph node (TdLN) 13 days after tumor inoculation.
(**D**) B78mCherryOVA cells were injected s.c. in the right flank of WT or *Clec9a*$^{gfp/gfp}$ mice. Percentage of mCherry$^+$ DC subsets in TdLNs, as analyzed by flow cytometry 13 days after injection of tumor cells.
(**E,F**) B16OVA tumors were inoculated s.c. in the right flank of WT or *Clec9a*$^{gfp/gfp}$ mice. At day 13 after inoculation, different intratumor cell populations (E) (TIDCs: tumor infiltrating DCs; TAMs: tumor associated macrophages) or TdLNs (F) were sorted. These cells were cocultured with CellViolet-labelled purified CD8$^+$ OTI cells at the indicated ratios. Proliferation of CD8$^+$ OTI cells was quantified by FACS by CellViolet dilution after 3 days.
(A) Pool of three independent experiments, with n=19 for each experimental group. (B) Pool of 2 independent experiments with n=14 and n=15 for WT and *Clec9a*$^{gfp/gfp}$ mice, respectively. (C,D) Pool of 2 independent experiments. Statistical significance was evaluated by Student's t test. (A-D) Each dot is a single mouse. (E,F) Pool of 3 (E) and 2 (F) experiments. Statistical significance was evaluated with two-way ANOVA. All data are shown as arithmetic mean ± SEM.

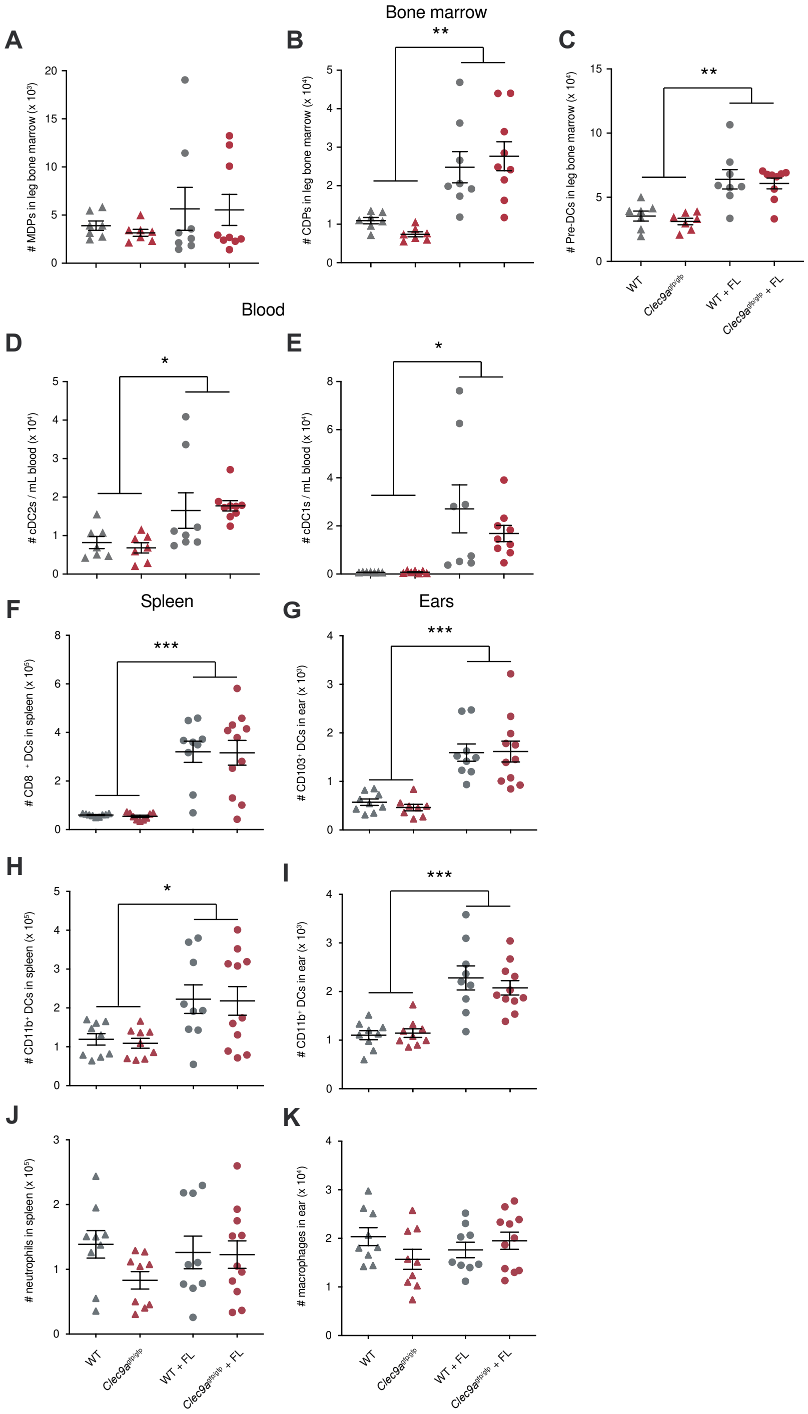


**Figure S3. Hydrodynamic i.v. inoculation of a secreted Flt3L-encoding plasmid expands populations of conventional dendritic cells.**

WT (in grey) and *Clec9a*$^{gfp/gfp}$ (in red) mice were hydrodynamically inoculated i.v. with a plasmid that encodes a secreted form of Flt3L (FL) or an empty control plasmid. 14 days later, cell suspensions from bone marrow, blood, spleen and skin were analyzed by flow cytometry.

(**A-C**) Numbers of MPDs (A), CDPs (B) and pre-DCs (C) in the bone marrow of femur and tibia from one leg. (**D-E**) Amount of cDC1s (D) and cDC2s (E) per mL blood.
(**F,H,J**) Numbers of $CD8^+$ (F), $CD11b^+$ (H) DCs and neutrophils (J) in the spleen.
(**G,I,K**) Numbers of $CD103^+$ (G), $CD11b^+$ (I) DCs and macrophages (K) in the ear skin.
Graphs show means ± SEM, where each dot represents a single mouse from a pool of 2 independent experiments. One-way ANOVA followed by Fisher's LSD test was used to evaluate the statistical significance. *$P < 0.05$, **$P < 0.01$ and ***$P < 0.001$.

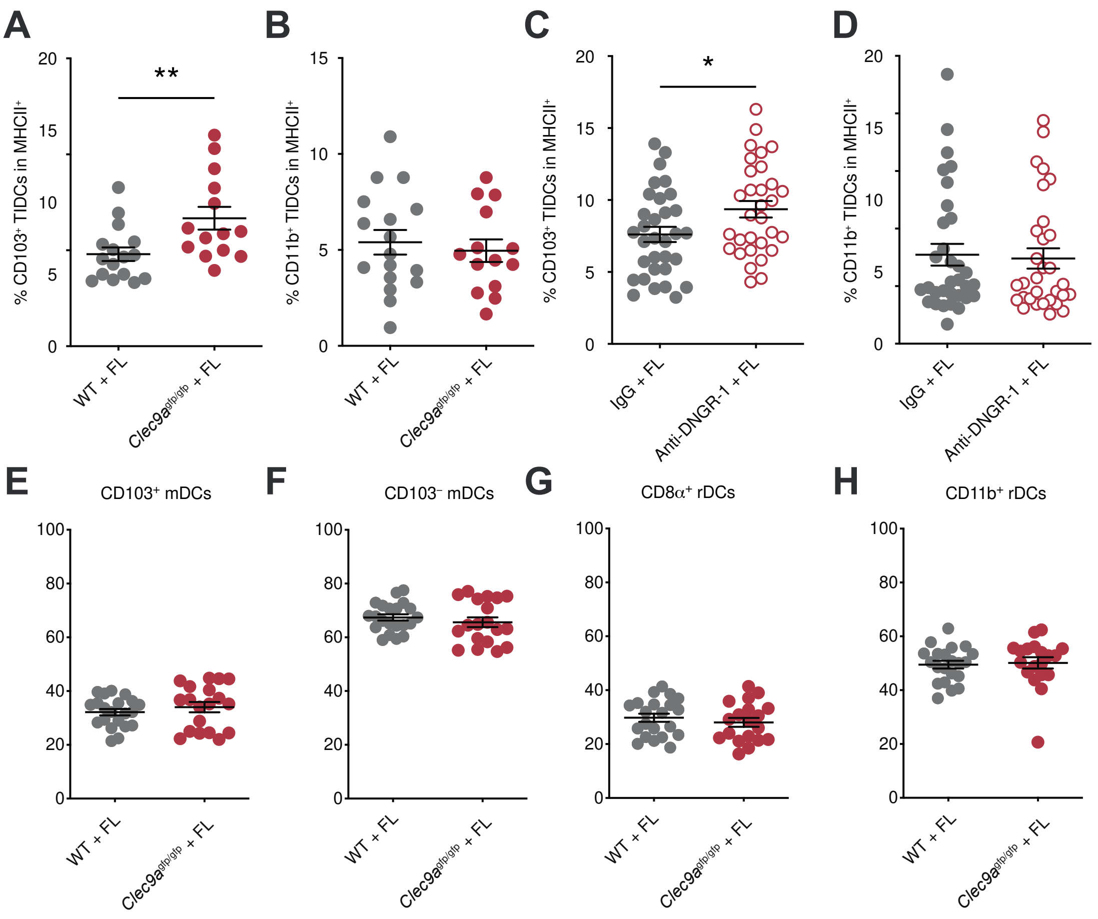


**Figure S4. Frequencies of dendritic cell subpopulations within the tumor microenvironment and tumor-draining lymph nodes.**

(**A,B**) WT and *Clec9a*$^{gfp/gfp}$ mice were treated with FL and inoculated, one day later, with B16F10 tumors in the right flank. 13 days later, tumor immune infiltrates were analyzed by flow cytometry and percentages of cDC1s (A) and cDC2s (B) within MHCII$^{+}$ cells were calculated.

(**C,D**) WT mice were inoculated with FL and challenged with B16F10 tumors one day later. Mice were treated with anti-DNGR-1 blocking antibodies or an isotype control at days 5, 7, 9 and 12 of tumor development. Frequencies of cDC1s (C) and cDC2s (D) within tumor-infiltrating MHCII$^{+}$ cells.

From the setting of (A,B), TdLNs were collected to evaluate their composition. (**E,F**) Percentage of cDC1s (E) and cDC2s (F) among migratory DCs. (**G,H**) Percentage of cDC1s (G) and cDC2s (H) among TdLN-resident DCs.

Graphs represents the mean ± SEM from a pool of 3 (A,B), 5 (C,D) and 3 (E-H) independent experiments, where each dot represents one mouse. Statistical significance was evaluated with Student's *t* test. $^{*}P < 0.05$ and $^{**}P < 0.01$

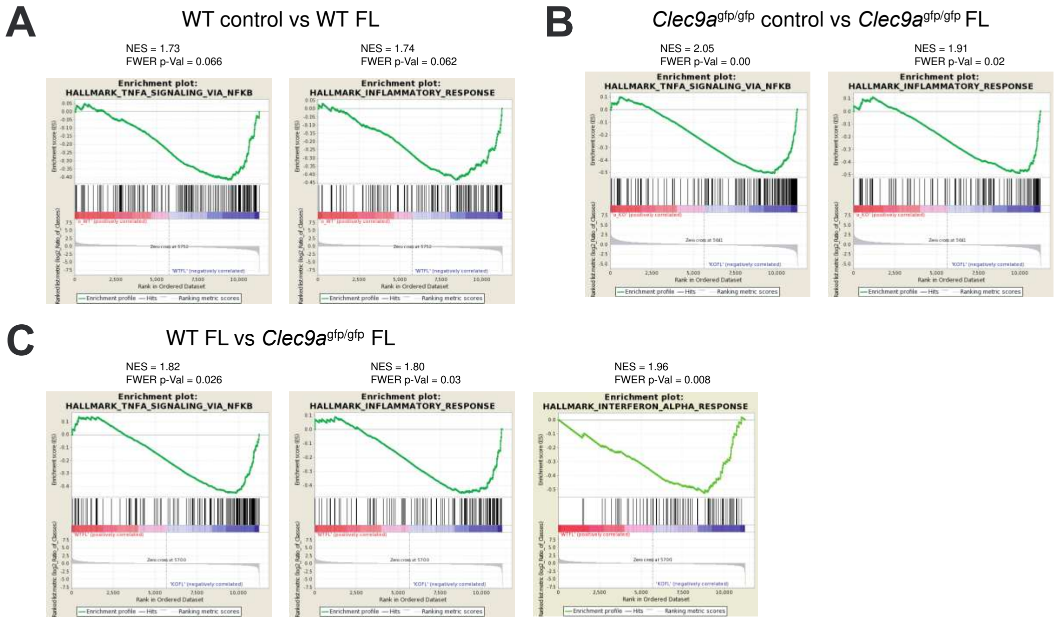


**Figure S5. Representative GSEA plots for gene sets.**

(**A**) Enrichment plots for gene sets enriched in cDC1s from WT mice inoculated with FL compared with the empty plasmid.

(**B**) Enrichment plots for gene sets enriched in *Clec9a*$^{gfp/gfp}$ cDC1s from mice treated with FL compared with an empty plasmid.

(**C**) Enrichment plots for gene sets enriched in *Clec9a*$^{gfp/gfp}$ cDC1s compared with WT cDC1s, both from mice treated with FL.

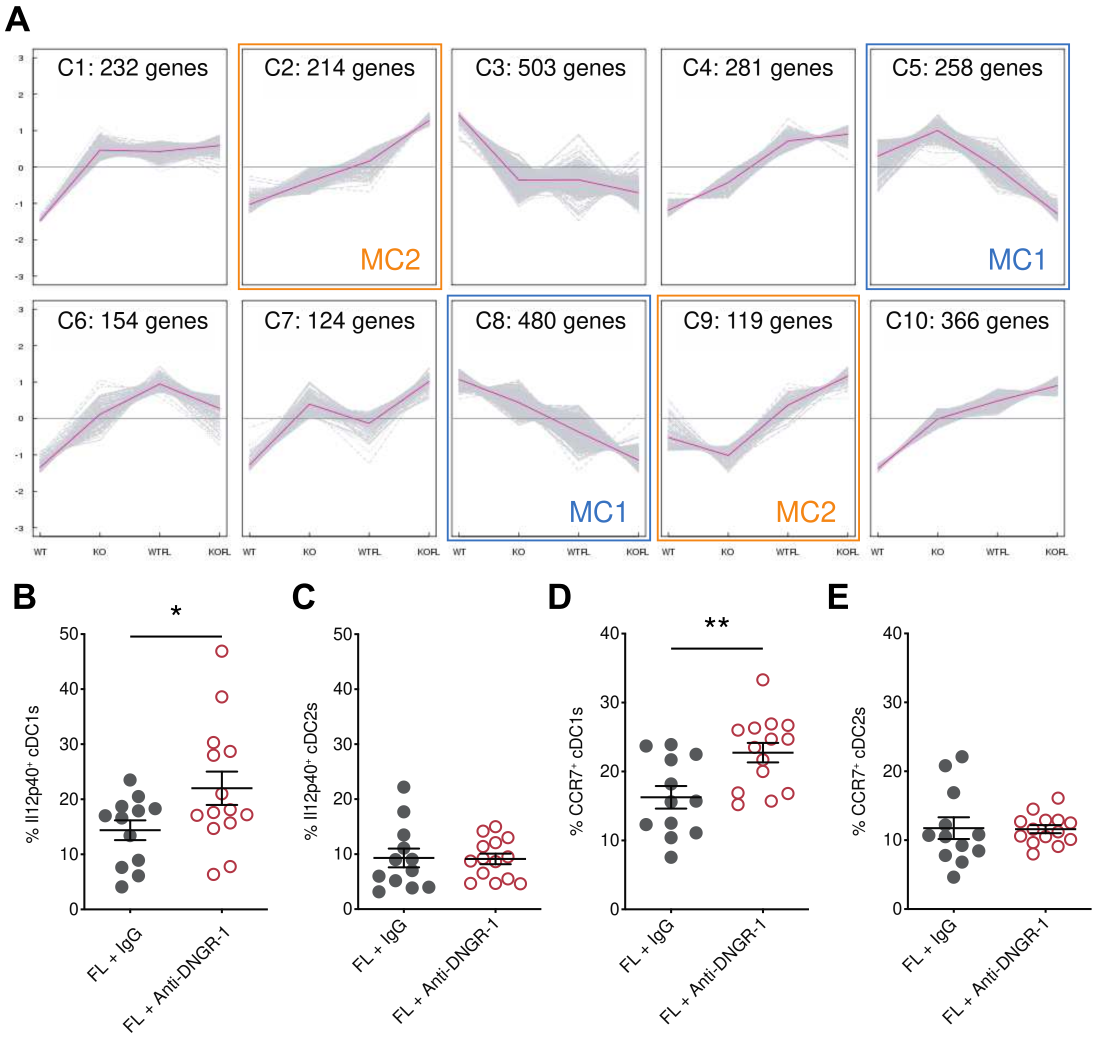


**Figure S6. K-means clusters of genes DE in at least one pairwise comparison.**

(**A**) Clusters 5 and 8 were grouped into metacluster 1 (MC1), composed of genes whose expression becomes lower in *Clec9a*$^{gfp/gfp}$compared with WT both treated with FL. Clusters 2 and 9 conform MC2, composed of genes whose expression becomes the highest in *Clec9a*$^{gfp/gfp}$ compared with WT upon FL treatment.

(**B-E**) Expression levels of IL12p40 (B,C) and CCR7 (D,E) on tumor-infiltrating cDC1s (B,D) and cDC2s (C,E) from FL-treated mice receiving control IgG or anti-DNGR-1 blocking antibody. Graphs show means ± SEM, where each dot represents a single mouse from a pool of 2 independent experiments. Student's *t* test was used to evaluate the statistical significance. *$P < 0.05$ and **$P < 0.01$.

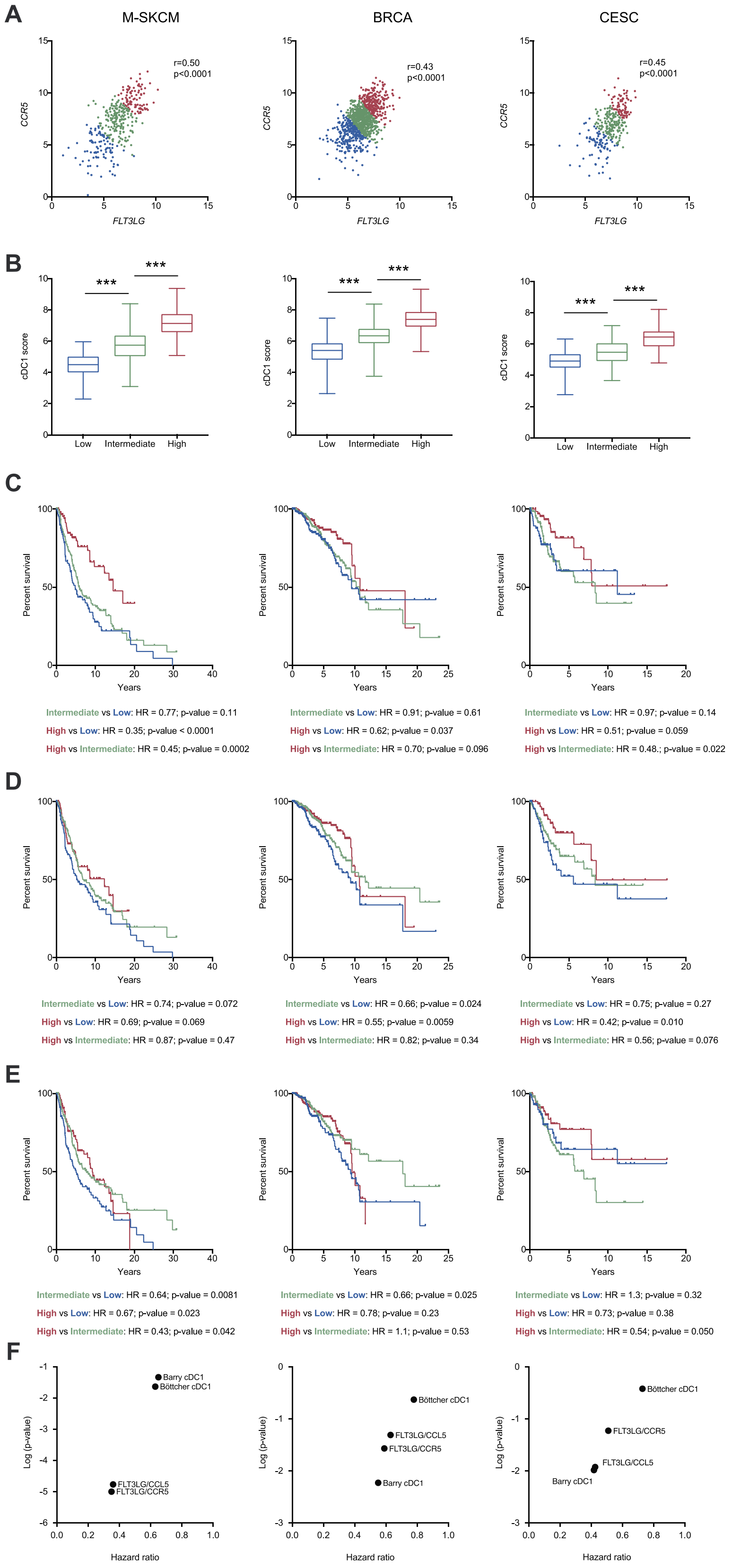
A
M-SKCM
BRCA
CESC
r=0.50
p<0.0001
r=0.43
p<0.0001
r=0.45
p<0.0001
CCR5
FLT3LG
B
cDC1 score
Low
Intermediate
High
***
C
Percent survival
Years
Intermediate vs Low: HR = 0.77; p-value = 0.11
High vs Low: HR = 0.35; p-value < 0.0001
High vs Intermediate: HR = 0.45; p-value = 0.0002
Intermediate vs Low: HR = 0.91; p-value = 0.61
High vs Low: HR = 0.62; p-value = 0.037
High vs Intermediate: HR = 0.70; p-value = 0.096
Intermediate vs Low: HR = 0.97; p-value = 0.14
High vs Low: HR = 0.51; p-value = 0.059
High vs Intermediate: HR = 0.48.; p-value = 0.022
D
Intermediate vs Low: HR = 0.74; p-value = 0.072
High vs Low: HR = 0.69; p-value = 0.069
High vs Intermediate: HR = 0.87; p-value = 0.47
Intermediate vs Low: HR = 0.66; p-value = 0.024
High vs Low: HR = 0.55; p-value = 0.0059
High vs Intermediate: HR = 0.82; p-value = 0.34
Intermediate vs Low: HR = 0.75; p-value = 0.27
High vs Low: HR = 0.42; p-value = 0.010
High vs Intermediate: HR = 0.56; p-value = 0.076
E
Intermediate vs Low: HR = 0.64; p-value = 0.0081
High vs Low: HR = 0.67; p-value = 0.023
High vs Intermediate: HR = 0.43; p-value = 0.042
Intermediate vs Low: HR = 0.66; p-value = 0.025
High vs Low: HR = 0.78; p-value = 0.23
High vs Intermediate: HR = 1.1; p-value = 0.53
Intermediate vs Low: HR = 1.3; p-value = 0.32
High vs Low: HR = 0.73; p-value = 0.38
High vs Intermediate: HR = 0.54; p-value = 0.050
F
Log (p-value)
Hazard ratio
Barry cDC1
Böttcher cDC1
FLT3LG/CCL5
FLT3LG/CCR5

**Figure S7. Cooccurrence of *FLT3LG* expression and the *CCR5* associates with increased cDC1 score and better survival in several human cancers.**

(**A**) Correlation of *FLT3LG* and *CCR5* expression within tumors of metastatic skin cutaneous melanoma (M-SKCM), breast carcinoma (BRCA) and cervical squamous carcinoma (CESC) using data extracted from the TCGA. Cancer patients were assigned a *FLT3LG*/*CCR5* score and sorted into low (first quartile), intermediate (second and third quartiles) and high (fourth quartile.

(**B**) cDC1 score based on expression of *KIT, CCR7, BATF3, FLT3, ZBTB46, IRF8, BTLA* and *MYCL1* as explained in methods was calculated within tumors from the quartile subcohorts of M-SKCM, BRCA and CESC patients.

(**C**) Overall survival of the quartile subcohorts of patients indicated in (A).

(**D, E**) cDC1 score was defined according to cDC1 signatures from Barry *et al* [9] and Böttcher *et al* [4] for the previously identified quartile subcohorts (A) of SKCM, BRCA and CESC patients. Overall survival of SKCM, BRCA and CESC patients from the quartile subcohorts described in (A).

(**F**) Representation of the hazard ratio and p-value of the comparison of groups high and low for the cDC1 signatures from Barry *et al* [9] and Böttcher *et al* [4] and the *FLT3LG*/*CCL5* and *FLT3LG/CCR5* signatures.

(A) Each point indicates a patient. r: Pearson correlation coefficient; p: p value. (B) Whisker plot of cDC1 score for cancer patients was calculated based on the expression of *KIT, CCR7, BATF3, FLT3, ZBTB46, IRF8, BTLA* and *MYCL1*. Statistical significance was assessed by one-way ANOVA followed by Fisher's LSD test. (C-F) Statistical significance of survival curves was assessed by pairwise Mantel-Cox test. ***$P<0.001$.
